# A Comprehensive Molecular Epidemiological Analysis of SARS-CoV-2 Infection in Cyprus from April 2020 to January 2021: Evidence of a Highly Polyphyletic and Evolving Epidemic

**DOI:** 10.3390/v13061098

**Published:** 2021-06-09

**Authors:** Andreas C. Chrysostomou, Bram Vrancken, George Koumbaris, George Themistokleous, Antonia Aristokleous, Christina Masia, Christina Eleftheriou, Costakis Iοannou, Dora C. Stylianou, Marios Ioannides, Panagiotis Petrou, Vasilis Georgiou, Amalia Hatziyianni, Philippe Lemey, Anne-Mieke Vandamme, Philippos P. Patsalis, Leondios G. Kostrikis

**Affiliations:** 1 Department of Biological Sciences, University of Cyprus, Aglantzia, Nicosia 2109, Cyprus; chrysostomou.c.andreas@ucy.ac.cy (A.C.C.); aristokleous.antonia@ucy.ac.cy (A.A.); stylianou.c.dora@ucy.ac.cy (D.C.S.); georgiou.a.vasilis@ucy.ac.cy (V.G.); 2 Department of Microbiology, Immunology and Transplantation, Rega Institute, KU Leuven, 3000 Leuven, Belgium; bram.vrancken@kuleuven.be (B.V.); philippe.lemey@kuleuven.be (P.L.); annemie.vandamme@uzleuven.be (A.-M.V.); 3 NIPD Genetics Limited, Nicosia 2409, Cyprus; g.koumbaris@nipd.com (G.K.); m.ioannides@nipd.com (M.I.); p.patsalis@nipd.com (P.P.P.); 4 Medical Laboratory of Ammochostos General Hospital, Ammochostos General Hospital, Paralimni 5386, Cyprus; themistokleousg1980@gmail.com (G.T.); christinamashiamm@gmail.com (C.M.); costasioannou0811@gmail.com (C.I.); ppetrou1982@gmail.com (P.P.); hatziyianniamalia@gmail.com (A.H.); 5 Department of Health and Safety, University of Cyprus, Aglantzia, Nicosia 2109, Cyprus; celeft03@ucy.ac.cy; 6 Center for Global Health and Tropical Medicine, Unidade de Microbiologia, Instituto de Higiene e Medicina Tropical, Universidade Nova de Lisboa, 1349-008 Lisbon, Portugal; 7 Medical School, University of Nicosia, Nicosia 2417, Cyprus

**Keywords:** SARS-CoV-2, Covid-19, Cyprus, molecular epidemiology

## Abstract

The spread of severe acute respiratory syndrome coronavirus 2 (SARS-CoV-2) resulted in an extraordinary global public health crisis. In early 2020, Cyprus, among other European countries, was affected by the SARS-CoV-2 epidemic and adopted lockdown measures in March 2020 to limit the initial outbreak on the island. In this study, we performed a comprehensive retrospective molecular epidemiological analysis (genetic, phylogenetic, phylodynamic and phylogeographic analyses) of SARS-CoV-2 isolates in Cyprus from April 2020 to January 2021, covering the first ten months of the SARS-CoV-2 infection epidemic on the island. The primary aim of this study was to assess the transmissibility of SARS-CoV-2 lineages in Cyprus. Whole SARS-CoV-2 genomic sequences were generated from 596 clinical samples (nasopharyngeal swabs) obtained from community-based diagnostic testing centers and hospitalized patients. The phylogenetic analyses revealed a total of 34 different lineages in Cyprus, with B.1.258, B.1.1.29, B.1.177, B.1.2, B.1 and B.1.1.7 (designated a Variant of Concern 202012/01, VOC) being the most prevalent lineages on the island during the study period. Phylodynamic analysis showed a highly dynamic epidemic of SARS-CoV-2 infection, with three consecutive surges characterized by specific lineages (B.1.1.29 from April to June 2020; B.1.258 from September 2020 to January 2021; and B.1.1.7 from December 2020 to January 2021). Genetic analysis of whole SARS-CoV-2 genomic sequences of the aforementioned lineages revealed the presence of mutations within the S protein (L18F, ΔH69/V70, S898F, ΔY144, S162G, A222V, N439K, N501Y, A570D, D614G, P681H, S982A and D1118H) that confer higher transmissibility and/or antibody escape (immune evasion) upon the virus. Phylogeographic analysis indicated that the majority of imports and exports were to and from the United Kingdom (UK), although many other regions/countries were identified (southeastern Asia, southern Europe, eastern Europe, Germany, Italy, Brazil, Chile, the USA, Denmark, the Czech Republic, Slovenia, Finland, Switzerland and Pakistan). Taken together, these findings demonstrate that the SARS-CoV-2 infection epidemic in Cyprus is being maintained by a continuous influx of lineages from many countries, resulting in the establishment of an ever-evolving and polyphyletic virus on the island.

## 1. Introduction

Severe acute respiratory syndrome coronavirus 2 (SARS-CoV-2) was discovered in December 2019 in Wuhan City, China (the capital of Hubei Province), and since then, has caused a pandemic [1]. The virus spread rapidly with unprecedented infectivity, especially in comparison to previous coronavirus epidemics, such as the severe acute respiratory syndrome (SARS) coronavirus (SARS-CoV) [2] and Middle East respiratory syndrome (MERS) epidemics [3]. This is evident from the fact that shortly after the start of the pandemic in January 2020, there were as many as 9927 cumulative cases of SARS-CoV-2 infections detected in at least 23 different countries/regions [4,5], and by the first quarter of 2021, there were more than 116,879,152 cases in 192 different countries/regions [4,5]. Significant factors that influenced the viral spread were the lack of herd immunity since methods such as vaccination against SARS-CoV-2 and effective pharmaceutical treatment/cure options had not yet been developed [6,7]. Thus, countries/regions opted to rely on policies such as social distancing, work closures, curfews, quarantine, travel and shopping restrictions to protect the population and prevent the collapse of health and economic systems [8,9]. These policies were implemented in conjunction with mass monitoring/screening of populations and contact tracing for SARS-CoV-2-positive individuals. However, such systems still place a heavy burden on the economy and are not permanent solutions [6]. Effective treatment and immunization methods are lacking [6,7], and disease prevention policies are mostly reactive to increases in SARS-CoV-2 cases, which facilitates a decrease in cases but does not prevent the spread of the virus. Thus, until the population is immunized and effective treatment is developed, it is important to optimize the current measures and policies [10].

This can be achieved by utilizing viral samples obtained from population monitoring and screening programs implemented by the government for the identification of SARS-CoV-2-infected individuals; analyzing and sequencing such samples provides the necessary basis for molecular epidemiological studies [11]. Molecular epidemiology has already provided significant insights into the evolution of this virus, and is currently being used to monitor/detect the accumulation of mutations in the viral genome [10,12]. It is important to note that SARS-CoV-2 is an RNA virus with proof-reading capabilities; however, it is evident that this is not enough to prevent the accumulation of mutations [13,14]. Furthermore, this virus has a global distribution, infecting populations of different genetic backgrounds, ages and health statuses, and it is subjected to evolutionary and selection pressures imposed by the host’s immune system, as well as by antiviral drugs [13,15,16]. Consequently, this results in the generation of viral lineages with slightly altered genetic make-ups and novel viral diversity in need of classification [17]. Although the SARS-CoV-2 nomenclature system has yet to be fully recognized, this study uses the viral lineage classification described by Rambaut et al. [17], who employed a phylogenetic framework to identify the lineages that contribute most to active spread [4,17,18]. Such classification systems are necessary for molecular epidemiological studies, since they enable the characterization of SARS-CoV-2 found in different population groups and niches, its diaspora, and its temporal dynamics and origins [19]. By identifying lineages varying in phenotype or antigenicity, health officials can remain alert and adequately prepare to safeguard public health [20].

Thus far, the evolution of SARS-CoV-2 has been continuously documented, with the S and L variants being reported at the end of February 2020 [21]; since then, its genetic variability has continued to expand. By March 2020, the D614G substitution, along with other accompanying mutations, started to sporadically appear, and by June 2020, it was prevalent enough to be present in over 74% of all published sequences [22]. Currently, there is a plethora of lineages with accumulated mutations circulating worldwide. In countries such as the UK, where the genetic diversity of the virus has been extensively described, the B.1.1.7 lineage emerged in September 2020 and is steadily increasing in prevalence [23]. Other lineages that have emerged around the world are B.1.351 and P.1, detected in October 2020 in South Africa and January 2021 in Japan/Brazil, respectively. While not limited to these three lineages, the mutations that these lineages harbor have been implicated in increased transmission and disease severity and/or decreased neutralization by sera from convalescent patients/vaccines [23].

In Cyprus, the nature of SARS-CoV-2 infection is still under investigation; hence, this work focuses on the molecular epidemiology of the virus in Cyprus to discern the prevalent lineages and the mutational landscape on the island. The first wave of the epidemic in Cyprus was adequately controlled due to the measures in place, and cases remained well below 100 per day [4,5,24]. However, in mid-October 2020, the number of cases began to rapidly increase, with more than 300 per day, ultimately reaching a peak of almost 1000 daily cases by the end of the sampling period in December. The number of cases then decreased (~100 daily cases) in early January 2021 [4,5,24].

In this study, we examined the molecular epidemiology of SARS-CoV-2 by analyzing samples from infected individuals in Cyprus from April 2020 to January 2021. We identified 34 different SARS-CoV-2 lineages in Cyprus, revealing a highly dynamic epidemic dominated by the B.1.1.29 lineage for the first three months of the study, and then by the B.1.258 lineage through the end of the sampling period; the UK strain B.1.1.7 appeared in the last two months of sampling. Furthermore, mutations/deletions in the S-protein, such as L18F, ΔH69/V70, S898F, ΔY144, S162G, A222V, N439K, N501Y, A570D, D614G, P681H, S982A and D1118H, were discovered in the lineages identified in this study and have phenotypic and antigenic implications that may impact the spread of the virus, as well as the efficiency of current vaccines and diagnostic tests. Moreover, we investigated the temporal and spatial aspects of SARS-CoV-2 in Cyprus and found that the majority of SARS-CoV-2 imports and exports were from and to the UK, although many other regions were also identified. These results allowed us to identify the lineages and mutations in Cyprus and to determine the effects of the measures implemented to protect public health.

## 2. Materials and Methods

### 2.1. Study Participants

From April 2020 to January 2021, 768 clinical nasopharyngeal samples were obtained from SARS-CoV-2-infected individuals from three cohorts in Cyprus. Specifically, 16 samples were obtained from the Laboratory of Biotechnology and Molecular Virology, University of Cyprus (BMV UCY), 502 samples were obtained from NIPD Genetics and 250 samples were obtained from Famagusta General Hospital (FGH). For the BMV UCY cohort, the 16 clinical nasopharyngeal samples were confirmed as SARS-CoV-2-positive by an in-house molecular-beacon-based real-time RT-PCR assay developed by the BMV UCY laboratory (manuscript in preparation for publication). For the NIPD Genetics cohort, the 502 samples were confirmed as SARS-CoV-2-positive by using the SensiFAST Probe No-ROX One-Step Kit (Meridian Bioscience, Cincinnati, OH, USA) and multiplex quantitative reverse transcriptase qPCR (RT-qPCR), and for the FGH cohort, the 250 samples were confirmed as SARS-CoV-2-positive using the SARS-CoV-2 Real-TM Kit for Real-Time PCR (Sacace Biotechnologies, Como, Italy). The nasopharyngeal samples from these individuals were retrospectively analyzed in this study after receiving approval by the Cyprus National Bioethics Committee (EEBK EΠ 2020.01.125, EEBK EΠ 2020.01.192). To ensure patient anonymity, all the samples were sent to the BMV UCY and coded with a laboratory or patient identification number. All the samples were further coded with a new laboratory identification number to ensure no connection of the samples to the corresponding study subjects could be made. The collection and use of the samples were in accordance with the relevant guidelines and regulations of the Cyprus National Bioethics Committee.

### 2.2. RNA Extraction and SARS-CoV-2 Real-Time RT-PCR

#### 2.2.1. BMV UCY Cohort

Nasopharyngeal samples were collected from students and personnel at the University of Cyprus using nasopharyngeal swabs (Biocomma, Shenzhen, China) and placed in Biocomma Virus and preservation medium tubes (Biocomma, Shenzhen, China). The tubes were then transported to the BMV UCY for automated RNA extraction using the QIAmp Viral RNA Mini Kit (Qiagen, Hilden, Germany) according to the manufacturer’s specifications for the QIAcube Connect (Qiagen, Hilden, Germany). The extracted RNA was tested for SARS-CoV-2 positivity using an in-house molecular-beacon-based real-time RT-PCR assay developed by the BMV UCY laboratory (manuscript in preparation for publication), which targets the SARS-CoV-2 structural S, E, M and N genes. Real-time RT-PCR analysis was completed using TaqPath™ 1-Step Multiplex Master Mix (No ROX) (Life Technologies, Frederick, MD, USA) on a 7900HT Fast Real-Time PCR System (Applied Biosystems, Foster City, USA).

#### 2.2.2. NIPD Cohort

Nasopharyngeal samples were collected by NIPD Genetics from people in Cyprus using nasopharyngeal swabs (Puritan, Guilford, ME, USA) and placed in 15 mL tubes (Thermo Scientific, Waltham, MA, USA) containing sterile viral transport medium. RNA was extracted using the TANBead OptiPure Viral Auto Plate Kit (TANBead, Taoyuan, Taiwan) on SLA-32 instrument according to the manufacturer’s instructions. Each RNA sample was then subjected to multiplex quantitative RT-qPCR using the SensiFAST Probe No-ROX One-Step Kit (Meridian Bioscience, Cincinnati, OH, USA) and two primer/probe sets targeting a conserved region of the SARS-CoV-2 N gene (N1 and N2) (Integrated DNA Technologies, Coralville, IA, USA).

#### 2.2.3. FGH Cohort

Nasopharyngeal samples were taken from patients at FGH using nasopharyngeal swabs and were placed in transport medium until further processing. RNA was extracted using the EZ1^®^ DSP Virus Kit (Qiagen, Hilden, Germany) according to the manufacturer’s specifications and a Qiagen EZ1 Advanced XL system (Qiagen, Hilden, Germany). SARS-CoV-2-positive samples were identified with the SARS-CoV-2 Real-TM Kit (Sacace Biotechnologies, Como, Italy) on the Qiagen Rotor-Gene Q MDx 5plex platform (Qiagen, Hilden, Germany).

### 2.3. Next Generation Sequencing (NGS)

#### 2.3.1. Library Preparation

Libraries for 768 SARS-CoV-2-positive RNA samples were prepared for sequencing following the Illumina Covidseq workflow (Illumina Inc., San Diego, CA USA) in accordance with the manufacturer’s specifications. The extracted RNA was reverse-transcribed to generate cDNA using random hexamers. Generated cDNA was amplified by dividing the cDNA into two pools according to the designed primers to produce 98 amplicons spanning the SARS-CoV-2 genome along with 11 control amplicons for human RNA. The two pools were then recombined to be simultaneously fragmented and tagmented. The tagmented amplicons were subjected to a post-tagmentation clean-up step and amplified once more with the addition of indexes to each sample using IDT for Illumina Nextera Unique Dual Indexes set A, B C and D (384 indexes for 384 samples). The indexed libraries were then pooled and cleaned in batches of 96 and quantified using the Qubit High-Sensitivity Assay on a Qubit 4.0 fluorometer (Invitrogen, Carlsbad, CA, USA). Each pool was then diluted to a concentration of 4 nM, and 25 μL from each pool was transferred into a new microcentrifuge tube to be denatured and then diluted as specified by the manufacturer of the NovaSeq 6000 SP flow cell workflow (Illumina Inc., San Diego, USA). The libraries were then loaded into the Illumina NovaSeq 6000 system for dual-indexed paired-end sequencing.

#### 2.3.2. Processing of Raw Sequencing Data

The sequencing data (bcl files) were demultiplexed using bcl2fastq conversion software [25]. The resulting paired-end fastq reads were aligned to the wuhCor1 assembly (NC_045512.2, GenBank: MN908947.3) using the Burrows-Wheeler transform algorithm [26]. The resulting BAM files were aligned with the wuhCor1 viral genome and then converted in a sequence alignment/map (SAM) alignment format using the mpileup algorithm of SAMtools [27] and the iVar algorithm [28] to generate consensus viral genomic sequences.

### 2.4. Bioinformatic Analysis

Data set compilation. The newly generated nearly whole genomes were multiple aligned using the MAFFT v.7.475 multiple sequence alignment program [29,30]. The alignments were visually inspected and manually edited with the AliView v.1.26 algorithm [31]. Editing allowed the removal of remaining putative sequencing artifacts (i.e., variations adjacent to gap regions that were not shared with other sequences). Furthermore, problematic sites, as previously defined [32], were subsequently stripped from the sequence alignment. A maximum likelihood (ML) tree was then estimated from the resulting alignment using IQTREE v.2.1.2 software [33], with branch support estimated through the SH-like approximate likelihood ratio test (SH-aLRT) [34]. To alleviate the computational burden in subsequent phylogenetic inference, the newly generated sequences were divided into six groups based on phylogeny. To infer time-scaled phylogeographic histories, the genomes from each subset were complemented with publicly available SARS-CoV-2 genomes. To this end, the available complete genomes (*n* = 537,361) were downloaded from GISAID on 16 February 2021 [4]. For each subset, the 25 most similar genomic sequences to each of the newly generated near-complete genomes were selected using the National Center for Biotechnology Information Basic Local Alignment Search Tool (NCBI BLASTn v.2.11.0+) [35], and all duplicate hits were removed. If identical sequences with the same country of origin were identified, only one was included to further reduce the dataset size. This procedure yielded datasets with 505 (B.1.258), 344 (B.1.177), 104 (B.1.1.7), 93 (B.1.2), 1146 (B.1.1.x) and 263 (B.basal) genomes. Data sets B.1.258, B.1.177, B.1.1.7, and B.1.2 were entirely composed of lineage B sequences, while B.1.1.x and B.basal contained 13 and one lineage A genome, respectively.

#### 2.4.1. Lineage Classification

The processed alignments were then input into Pangolin software v.2.3.3 to determine the lineage classification for each sample [17,36].

#### 2.4.2. Mutation Calling

Mutations of the sequences in this study were identified using the Nextclade webtool (https://clades.nextstrain.org/, accessed on 15 February 2021) [18].

#### 2.4.3. Phylogenetic, Phylodynamic and Phylogeographic Inferences

The presence of a sufficient temporal signal was investigated using TempEst cross-platform software [37]. The Bayesian Evolutionary Analysis Sampling Trees software package (BEAST v.1.10) [38] was used to infer time-scaled evolutionary histories from each data subset. An HKY nucleotide substitution model with gamma-distributed rate variation among sites was specified along with a strict molecular clock. A normal distribution (with mean and standard deviation of 0.0008 and 0.0001, respectively) was specified for the evolutionary rate parameter. For datasets B.1.258, B.1.177, B.1.1.7 and B.1.2 (entirely composed of lineage B genomes), the root height was constrained to be more recent than 1 January 2020. For the datasets with mixed lineage A and B genomes (B.1.1.x and B.basal), this constraint was set to 1 December 2019, while the lineage B taxa were also constrained to be monophyletic. Uncertainty in the tip dates was accommodated by integrating the sampling dates over appropriate intervals [39] using uniform priors. Whenever possible, the sampling date interval was narrowed using the date of submission to the Global Initiative on Sharing all Influenza Data (GISAID) database (e.g., when 2020 was specified as the sampling date but the submission date was prior to 31 December 2020, the latter was used as the upper bound of the sampling time uncertainty). A skygrid Bayesian nonparametric model [40] with 2 weekly change points ranging from 15 January 2021, to the time to the most recent common ancestor (tMRCA) constraint was used as a flexible tree topology prior.

Migration history was inferred using an asymmetric discrete phylogeographic model that incorporated a model averaging procedure (the Bayesian stochastic search variable selection procedure [41]) to identify subsets of migration flows that adequately explained the diffusion process [42]. We accounted for only migration links with a Bayes factor ≥ 5. The expected number and timing of transitions between locations was estimated using stochastic mapping techniques [43]. For all datasets except the B.1.1.x dataset, the location was set to the reported country of origin, and the location trait history was inferred simultaneously with sequence evolution.

The size and complexity of the B.1.1.x dataset led us to use an alternative approach for this dataset to accommodate phylogenetic uncertainty in the migration history reconstruction. Here, we first inferred a set of plausible evolutionary histories (using the aforementioned model specifications) that was subsequently used as an empirical tree distribution [44] to which the location trait history was fitted. This assumes that the likelihood of a tree topology is dominated by the variation among sequences in the alignment. However, because of the limited diversity among SARS-CoV-2 genomes, the location trait can exert a nonnegligible influence. For this reason, a simplified phylogeographic model in which all non-Cypriot taxa were binned by region as defined by GISAID was also specified to obtain the empirical tree distribution. B.1.1.x genomes were sampled in 68 countries; however, many were represented by only a single taxon or a few taxa (Table S1). To reduce the complexity of the migration model, we opted to group genomes from countries with fewer than 20 genomes by United Nations geographical subregion. The number of location states was further reduced from 31 to 23 by grouping the genomes from Melanesia and Micronesia as Oceania (*n* = 2 taxa) and by assigning Africa as the sampling location of the 14 genomes from eight African countries in this dataset.

The convergence and mixing properties of the Markov chains were assessed with Tracer v1.7 [45], and post-burn-in samples of several independent chains were combined when needed. Maximum clade credibility trees summarizing the (combined) post-burn-in MCMC samples were generated with TreeAnnotator software v1.10.5.

## 3. Results

### 3.1. The Appearance of SARS-CoV-2 Lineages in Cyprus

**Figure 1:**
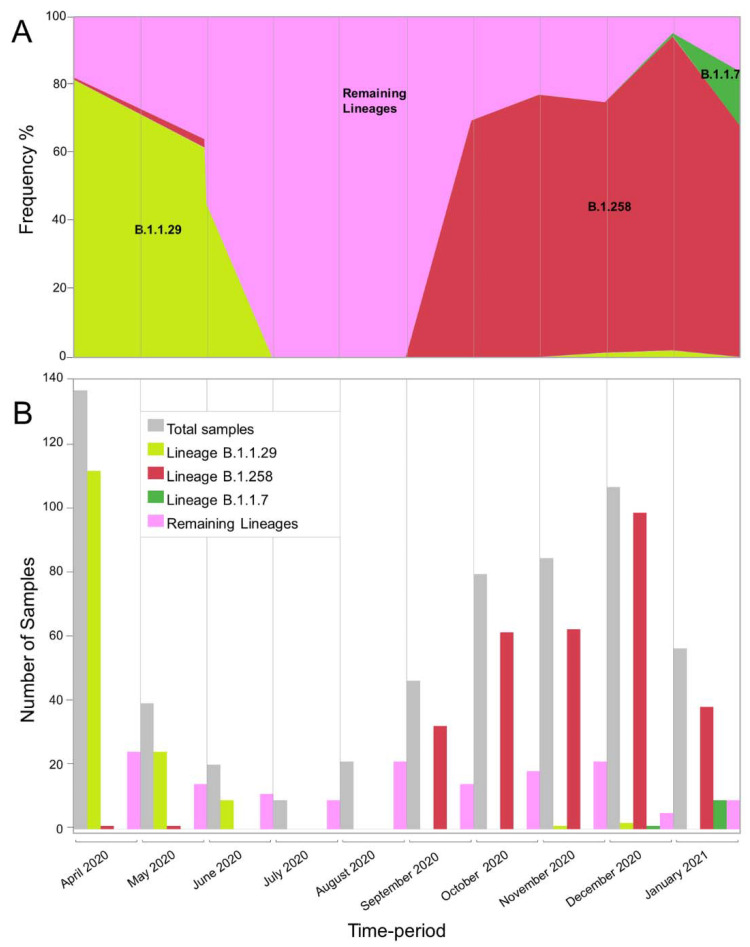
Graphical representation of the appearance of lineages B.1.1.29, B.1.258 and B.1.1.7 in each month of the sampling period from April 2020 to January 2021. (**A**) The frequency of the two most prevalent lineages, as well as B.1.1.7, over the 10-month sampling period. The *y*-axis represents the frequency of samples as percentages, while the x-axis represents the time-period of each month of the sampling period. The samples for lineages B.1.1.29, B.1.258, and B.1.1.7 are shown in light green, red and green, respectively. The numbers of samples in the remaining lineages are shown in pink and were calculated by excluding the monthly samples in the B.1.1.29, B.1.258, and B.1.1.7 lineages from the total number of samples (Table 1). (**B**) Bar chart indicating the number of samples of the two most prevalent lineages, as well as B.1.1.7, over the 10-month sampling period. The y-axis represents the number of samples that were identified in each month of the sampling period. The total number of samples is shown in gray (indicated in Table 1), and the samples belonging to lineages B.1.1.29, B.1.258, and B.1.1.7 are shown in light green, red and green, respectively. The numbers of samples in the remaining lineages are shown in pink and were calculated by excluding the monthly B.1.1.29, B.1.258, and B.1.1.7 lineage samples from the total number of samples (Table 1). The *x*-axis represents each month in the sampling period.

In this study, 596 SARS-CoV-2 whole-genome sequences were analyzed from infected people in Cyprus. The sampling period was from April 2020 to January 2021. Analyzing these sequences with Pangolin software [36] revealed that there were 34 different lineages in this dataset (Table 1). In Table 1, the appearances of these sequences are shown; the first six months of the sampling period are stratified into three months intervals, and the last four months are stratified into two-month intervals. During the first period (April–June 2020), the dataset was dominated by the B.1.1.29 lineage, accounting for ~74% of the sequences (144/195) (Figure 1). There was a large difference from the second most prominent lineage of that period, B.1, accounting for only ~9% (17/195) of the total sequences. During the second period (July–September 2020), no B.1.1.29 sequences were detected. The July–September 2020 period was characterized by the lowest number of sequences, with a total of 76, in contrast to the other three periods (April–June 2020, October–November 2020, and December 2020–January 2021), which all had more than 160 sequences. The most prominent lineages during the July–September 2020 period were B.1.258, accounting for ~42% (32/76), and B.1.2, accounting for ~24% (18/76). From that point on, the prevalence of B.1.258 continued to increase, accounting for the vast majority of sequences in the last two periods, at ~75% (123/163, October–November 2020) and ~84% (136/162, December 2020–January 2021) (Figure 1). The B.1.177 lineage was identified much less frequently during these two periods. Notably, the last month of sampling, January 2021 (Figure 1), which was dominated by the B.1.258 lineage, marked the appearance of the UK strain B.1.1.7 on the island. This strain is represented in this dataset with 10 sequences.

### 3.2. Mutational Analysis of the S Protein to Identify the Most Prevalent Lineages in Cyprus

**Figure 2:**
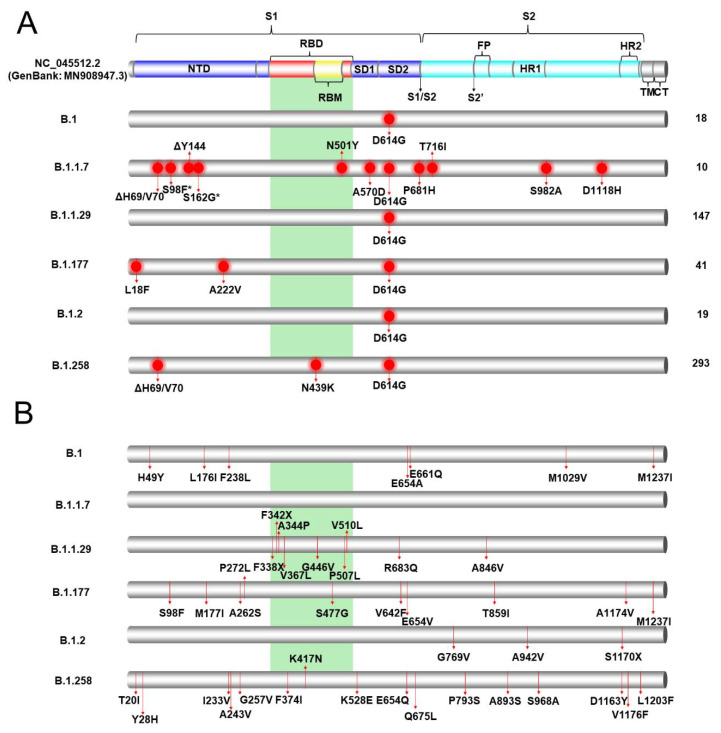
Common and uncommon mutations/deletions identified in the S proteins of the most prevalent lineages (B.1.258, B.1.1.29, B.1.177, B.1.2, B.1 and B.1.1.7) in Cyprus. The colored cylinder depicts key domains of the SARS-CoV-2 S protein (NC_045512.2) (GenBank: MN908947.3). NTD, N-terminal domain; RBD, receptor-binding domain (red); RBM, receptor-binding motif (yellow); SD1, subdomain 1; SD2, subdomain 2; FP, fusion peptide; S1, subunit 1 (blue); S2, subunit 2 (cyan); HR, heptad repeats; TM, transmembrane domain (gray); CT, cytoplasmic tail (gray). The cleavage sites are depicted with black arrows, and their respective titles are S1/S2 and S2′. The green highlighted region corresponds to the receptor binding domain (RBM). Since this field is still under global scientific investigation, a number of published sources were used to identify each domain along with their start and end locations [50,51,52,53,54,55,56,57,58,59]. The mutations/deletions were identified using the Nextclade webtool [16]. (**A**) Common mutations/deletions in each lineage. Red circles indicate the locations of common mutations/deletions that were identified in all or nearly all of the sequences in a lineage. Asterisks represent mutations/deletions that were detected in approximately half of the sequences in the B.1.1.7 lineage; S98F was found in 5/10 sequences, and S162G was found in 4/10 sequences. On the right-hand side of the figure, the total number of sequences per lineage in this study is indicated. (**B**) Uncommon mutations/deletions in each lineage. Red lines indicate the locations of uncommon mutations/deletions that were identified in at least one sequence per lineage. To retain the clarity of the figure, for B.1.1.29 and B.1.258, only the mutations/deletions found in the RBD or in at least two sequences are indicated. The full list of common and uncommon mutations/deletions is indicated in the supporting information (Tables S2 and S3).

The six most prevalent lineages identified in Cyprus in the current study were B.1.258 49.2% (293/596), B.1.1.29 24.7% (147/596), B.1.177 6.8% (41/596), B.1.2 3.2% (19/596), B.1 3.0% (18/596) and 1.7% B.1.1.7 (10/596) (Table 1); hence, these lineages were selected for the mutational analysis described in this section, as well as the analyses that follow. Mutational analysis was focused on the S protein due to its importance in current vaccines and diagnostic assays [46]. To perform this analysis, the sequences were input into the nextclade webtool [18], and the output data were sorted to isolate S protein mutations and deletions. The common mutations that were identified in the sequences of each lineage are presented in Figure 2A and Table S2. The lineage with the most mutations/deletions, which were common in all the S protein sequences in the lineages, was B.1.1.7, with 12 mutations/deletions. These mutations/deletions were ΔH69/V70, S98F, ΔY144, S162G, N501Y, A570D, D614G, P681H, T716I, S982A and D1118H. The sequences of lineage B.1.177 contained L18F, A222V and D614G mutations, and those of lineage B.1.258 contained ΔH69/V70, N439K and D614G mutations/deletions. The D614G mutation was present in essentially all the sequences of all six lineages, and it was the only common mutation found in lineages B.1, B.1.1.29 and B.1.2. ΔH69/V70 mutations were found in only lineages B.1.1.7 and B.1.258. These two were the only lineages with common mutations within the receptor-binding domain (RBD) of the S protein. Most mutations found in all six lineages were concentrated on the S1 subunit of the S protein; however, in the B.1.1.7 lineage, mutations such as S982A and D1118H were identified on the S2 subunit.

**Figure 3:**
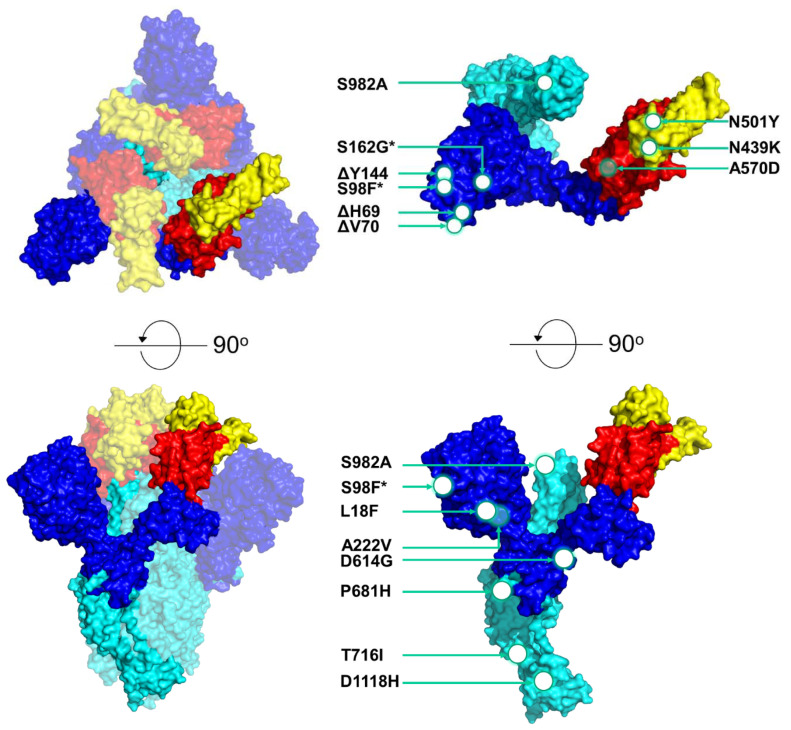
3D schematic model of the SARS-CoV-2 S protein showing the locations of common mutations/deletions in the most prevalent lineages (B.1.258, B.1.1.29, B.1.177, B.1.2, B.1 and B.1.1.7) in Cyprus. The model was produced by PyMol (Version 2.4.1, Schrödinger, LLC, https://www.pymol.org, accessed on 18 February 2021) and is based on data derived and adapted from Protein Data Bank entry 6XEY [49]. On the left side, the S protein trimer is shown as a transparent surface representation (transparency 0.5), but the S protein monomer is not transparent. On the upper-left side of the figure, the top of the protein is depicted, and on the lower-left side, the S protein has been rotated at a 90-degree angle to show the side view. On the right side of the figure, the S protein monomer is depicted in the upper right showing the top view, and the lower right side shows the side view of the monomer. Circles and arrows indicate the approximate locations of the common S-protein mutations/deletions identified in the most prevalent lineages (B.1.258, B.1.1.29, B.1.177, B.1.2, B.1 and B.1.1.7) in Cyprus in this study. White circles represent mutations/deletions on the outer surface, while open circles represent mutations within the protein or on the backside of the protein. Asterisks represent mutations/deletions that were found in approximately half of the sequences in the B.1.1.7 lineage; S98F was found in 5/10 sequences, and S162G was found in 4/10 sequences. The colored domains are RBDs. RBD, receptor-binding domain (red); RBM, receptor-binding motif (yellow); SD1, subdomain 1; SD2, subdomain 2; FP, fusion peptide; S1, subunit 1 (blue); S2, subunit 2 (cyan). Since this field is still under global scientific investigation, a number of published sources, including the UniProt entry P0DTC2, were used to identify each domain along with their start and end locations [50,51,52,53,54,55,56,57,58,59].

In Figure 2B, the uncommon mutations in the six most prominent lineages can be observed. For the purposes of this study, uncommon mutations were defined as polymorphisms represented in at least one sequence and in less than half of the sequences of a lineage. In lineage B.1.1.7, there were no uncommon mutations, which is in contrast with lineage B.1.1.29, which had 21 mutations and seven deletions, and to lineage B.1.258, which had 29 mutations (Table S3). Due to the large numbers of polymorphisms in lineages B.1.1.29 and B.1.258, only the mutations/deletions found in the RBD or in at least two sequences were indicated. Contrary to Figure 2A, in these six lineages, there was a larger number of mutations found in the S2 subunit and in the RBD. In lineage B.1.1.29, F338X, F342X, A344P, V367L, G446V, P507L and V510L were each represented in a different sequence, and in lineage B.1.177, only the S477G mutation was found in the RBD in only one sequence. In the B.1.258 lineage, three mutations, F374I, K417N and K528E, were detected in the RBD and were found in different sequences. Interestingly, the K417N mutation was reported to be one of the lineage-defining mutations of South African lineage B.1.351 [47,48]. In Figure 3, the most common mutations in the six most prevalent lineages in this study are depicted on a 3D-annotated model of the S protein. Figure 3 was developed based on data derived and adapted from Protein Data Bank entry 6XEY [49].

### 3.3. Phylogenetic Analysis of Cypriot SARS-CoV-2-Infected Individuals

**Figure 4:**
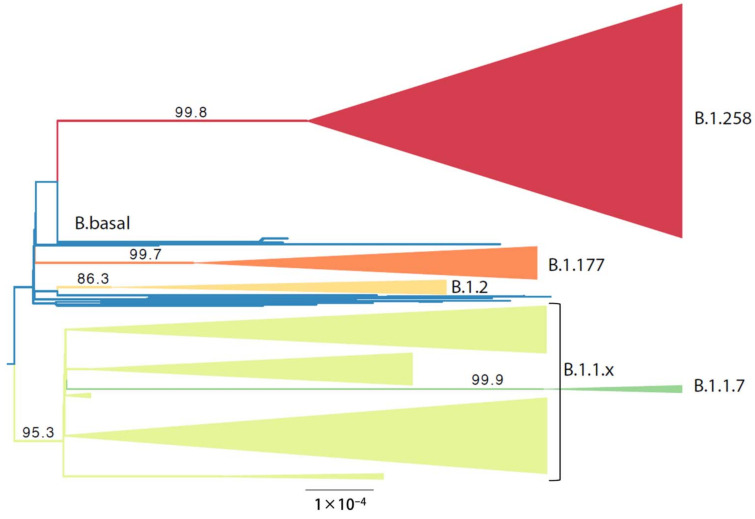
The maximum likelihood phylogeny used to delineate lineage-specific subsets among the newly generated Cypriot SARS-CoV-2 complete genomes. The tree is midpoint rooted. Branch support values are indicated next to the relevant branches. The B.1.258 clade is represented by red, the B.basal clade by blue, the B.1.177 clade by orange, the B.1.1.x clade by light green and the B.1.1.7 clade by green. The scale is provided by the black bar below the tree in substitutions per site.

The analysis of the Cypriot SARS-CoV-2 sequences to discern their genetic relationship started with the construction of a maximum likelihood (ML) tree that also served to guide the delineation of relevant subsets. The ML tree was estimated from nearly whole Cypriot genomes (Figure 4). In this phylogenetic analysis, six groups that largely corresponded to specific lineages, as identified by Pangolin software [36], were identified. All taxa in clades B.1.258 (*n* = 294), B.1.2 (*n* = 19), B.1.1.7 (*n* = 10) and B.1.177 (*n* = 42) were assigned by Pangolin to the lineage that was used to name the subset. Clade B.1.1.x encompasses 223 taxa, the majority of which are assigned to the B.1.1.29 lineage by Pangolin (*n* = 147). Within clade B.1.1.x, excluding B.1.1.29, the following lineages were identified: A (*n* = 1), B (*n* = 5), B.1 (*n* = 13), B.1.1.1 (*n* = 7), B.1.1.130 (*n* = 1), B.1.1.131 (*n* = 2), B.1.1.141 (*n* = 2), B.1.1.153 (*n* = 3), B.1.1.159 (*n* = 2), B.1.1.161 (*n* = 5), B.1.1.192 (*n* = 2), B.1.1.218 (*n* = 2), B.1.1.230 (*n* = 1), B.1.1.251 (*n* = 4), B.1.1.277 (*n* = 4), B.1.1.288 (*n* = 2), B.1.1.307 (*n* = 1), B.1.1.315 (*n* = 1), B.1.1.317 (*n* = 2), B.1.1.41 (*n* = 4), 1 B.1.1.67 (*n* = 1), B.6 (*n* = 1). Lineage B.1.1.7 (*n* = 10) was also included in clade B.1.1.x; however, it was isolated and subjected to further analysis. The last subset contains 17 taxa that cluster basally to the aforementioned groups, to which we refer as B.basal. The composition of the B.basal clade consists of the following lineages: B.1 (*n* = 5), B.1.160 (*n* = 2), B.1.236 (*n* = 6), B.1.313 (*n* = 2), and B.1.36 (*n* = 2).

### 3.4. Timed Migration Histories

Phylodynamic and phylogeographic analyses were performed to estimate the timed migration histories of the sequences in this study. First, the evolutionary rates of each of the clades, as shown in Figure 4, were estimated by investigating the presence of temporal signals through the regression of sampling time to root-to-tip genetic distance (Figure S1). This analysis indicated that none of the estimated evolutionary rates calculated in this study was in line with the estimated long-term SARS-CoV-2 evolutionary rate of approximately 0.0008 substitutions/site/year (s/s/y) [60]. For this reason, the normal distribution, with a mean equal to 0.0008 and a standard deviation of 95% of the density of the prior distribution, which is between 0.0006 and 0.001 s/s/y, was specified as a prior in the evolutionary rate parameter for inferring time-scaled phylogeographic histories. Note that 0.0008 s/s/y was also used as the evolutionary rate in simulations by Worobey et al. [61].

The timing of the first import event for the different datasets of SARS-CoV-2 into Cyprus was estimated at (in chronological order based on the mean estimate): 20 January 2020 (B.1.1.x, 95% HPD: 2 January 2020–5 February 2020), 12 February 2020 (B.basal, 95% HPD: 21 December 2019–9 March 2020), 12 March 2020 (B.1.177, 95% HPD: 2 February 2020–8 April 2020), 31 March 2020 (B.1.2, 95% HPD: 31 January 2020–22 July 2020), 21 April 2020 (B.1.258, 95% HPD: 3 January 2020–26 October 2020), and 1 December 2020 (B.1.1.7, 95% HPD: 18 November 2020–19 December 2020).

**Figure 5:**
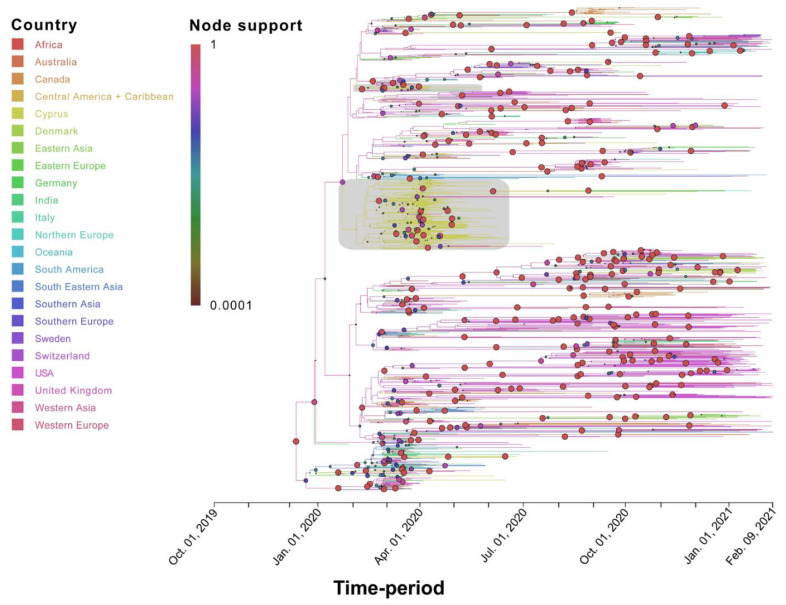
Time-scaled migration history for the B.1.1.x dataset. The link between branch color and the inferred location (Country) is shown in the top left of the figure. The size and color of the circles at the nodes (representing inferred common ancestors) indicate their posterior support (node support); the level of node support (highest = 1 and lowest = 0.0001) corresponds to the size of the circle (higher support = larger circle size). The link between color and posterior node support is shown in the top left of the figure. The gray-shaded boxes indicate two clades in the MCC summary tree that consist almost uniquely of Cypriot taxa detected in April and May 2020. The *x*-axis representing the time-period of the reconstruction was generated by the maximum clade credibility summary tree.

**Figure 6:**
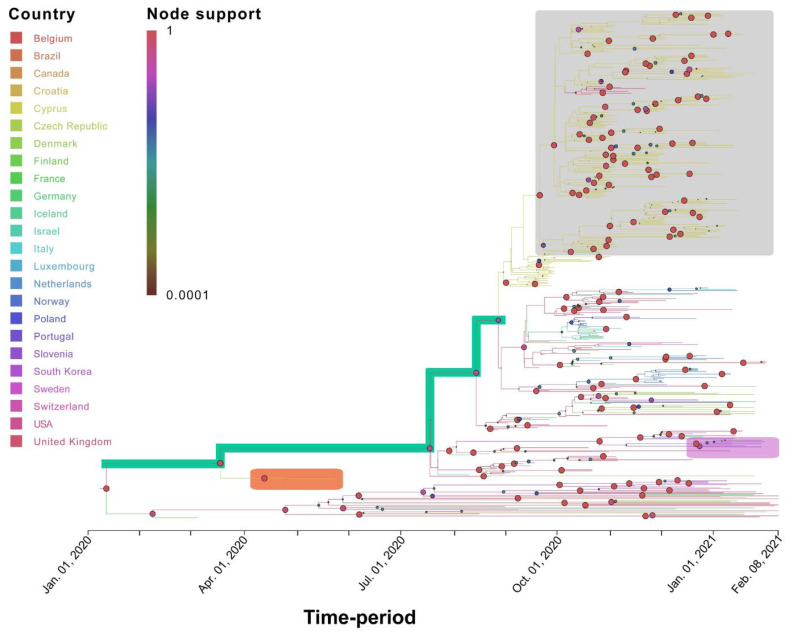
Time-scaled migration history for the B.1.258 dataset. The link between branch color and the inferred location (Country) is shown in the top left of the figure. The size and color of the circles at the nodes (representing inferred common ancestors) indicate their posterior support (node support); the level of node support (highest = 1 and lowest = 0.0001) corresponds to the size of the circle (higher support = larger circle size). The link between color and posterior node support is shown in the top left of the figure. The gray-shaded box indicates the well-supported B.1.258 Cyprus-specific lineage. The orange-shaded box indicates the two earliest Cypriot B.1.258 samples. The green-shaded branches indicate the backbone lineages. The pink-shaded box indicates the Cypriot sample that clustered among isolates from Slovenia and Switzerland. The *x*-axis represents the time-period of the reconstruction and was generated by the maximum clade credibility summary tree.

**Figure 7:**
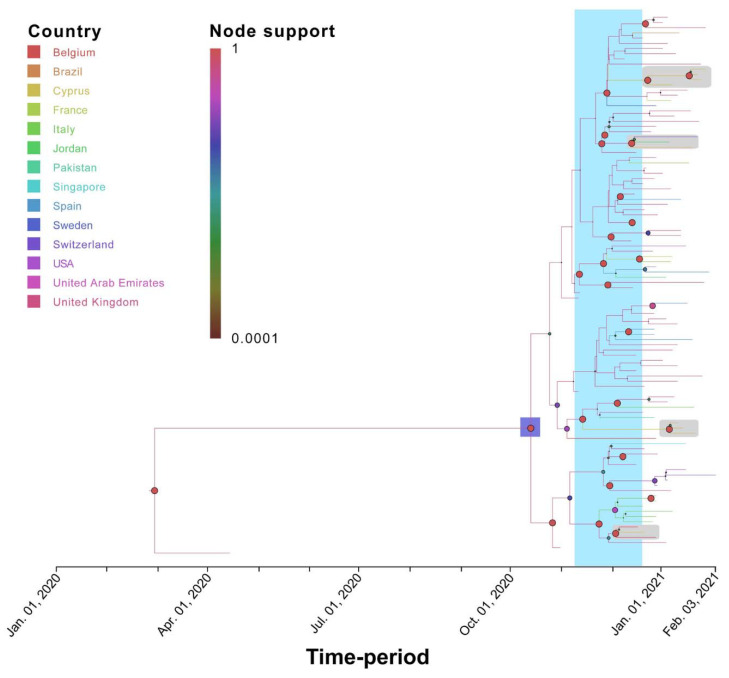
Time-scaled migration history for the B.1.1.7 dataset. The link between branch color and the inferred location (Country) is shown in the top left of the figure. The size and color of the circles at the nodes (representing inferred common ancestors) indicate their posterior support (node support); the level of node support (highest = 1 and lowest = 0.0001) corresponds to the size of the circle (higher support = larger circle size). The link between color and posterior node support is shown in the top left of the figure. The purple-shaded area represents the 95% HPD of the B.1.1.7 tMRCA. The light blue-shaded area represents the time of first introduction of B.1.1.7 into Cyprus. The gray-shaded boxes indicate the Cypriot samples. The *x*-axis representing the time-period of the reconstruction was generated by the maximum clade credibility summary tree.

Specifically, for the most prevalent lineages, B.1.1.29 (April–June 2020) and B.1.258 (September–January 2020), as well as the UK lineage B.1.1.7, which appeared in December and subsequently increased in prevalence, timed migration analyses revealed their temporal and spatial dynamics in Cyprus (Figure 1 and Figure 5, Figure 6 and Figure 7). The B.1.1.29 lineage comprised the majority of the taxa of the B.1.1.x clade, accounting for 69.0% (147/213) (Figure 5). Most B.1.1.29 samples were collected from April to June 2020, accounting for ~98% (144/147 total B.1.1.29 samples) (Table 1 and Figure 1). The timed evolutionary reconstructions showed that after the first import event, estimated to be 20 January 2020 (B.1.1.x, 95% HPD: 2 January 2020–5 February 2020), Cypriot B.1.1.29 samples had few progeny, indicating that they were mostly imports that did not propagate (Figure 5, gray-shaded boxes). However, for the lineage that subsequently became prevalent, B.1.258, the timed evolutionary reconstructions indicate an uncertainty that extended beyond the date of sampling of the two earliest isolates, which cluster as a sister lineage to isolates from other countries, with high support (Figure 6, orange-shaded box). In the most plausible ancestral reconstruction, the backbone lineages leading to the formation of this early Cypriot clade are estimated to have originated in the UK, meaning that the date of the first introduction should be constrained by the first sampling date. The origin associated with the first jump of this lineage towards Cyprus is either the UK (87% of the reconstructions) or Slovenia (13% of the reconstructions) according to our reconstructions. The latter signal arose from a single genome from Cyprus that clustered with four genomes obtained in Slovenia and one in Switzerland (Figure 6, pink-shaded box) with perfect support, with Cyprus as the location of the backbone lineages (Figure 6, green-shaded branches). However, we deemed the aforementioned scenario of Cyprus as the origin of the B.1.258 lineage to be highly unlikely. First, this would imply that B.1.258 variants circulating in Cyprus were not detected in the 61 successfully sequenced samples collected during the nearly four-month period between 25 May 2020 and 21 September 2020, which were the sampling dates of the first and last B.1.258 samples collected before and after the summer period. Second, this would imply that Cyprus was the origin of the global spread of variants in this clade, which was estimated to have started in early January (8 January 2020, 95% HPD: 1–21 January 2020), and likely even earlier if it were not for the tMRCA constraint (Figure S2). This corresponds to a start of spread before the establishment of the first sustained transmission network in Europe (Italy, 28 January 2020, 95% HPD: 20 January 2020–6 February 2020 [61]) and suggests nondetection among the first 129 successfully sequenced samples through 26 February 2020. Cyprus as the origin of this clade in our reconstructions with nonnegligible probability was deemed to be the result of the uneven availability of SARS-CoV-2 genomes from different countries. Therefore, the more likely date of introduction of B.1.258 in Cyprus, estimated by alternative reconstructions, is 7 March 2020 (95% HPD: 17 January 2020–16 April 2020). For the B.1.1.7 lineage, which started to increase in prevalence in Cyprus in December 2020 and January 2021, the MRCA dates to 14 October 2020 (95% HPD: 28 September 2020–30 October 2020) (Figure 7, purple-shaded area). This leads to the conclusion that this lineage was first imported into Cyprus 40 to 82 days after its genesis (Figure 7, light blue-shaded area) [62,63]. Furthermore, the Cypriot B.1.1.7 samples shown in the gray-shaded boxes (Figure 7) do not cluster in the same branches, indicating different subclusters that originated from different MRCAs. It is important to note that although not as prevalent as the B.1.1.29 and B.1.258 lineages, the B.1.1, B.1.2 and B.1.177 lineages were prevalent (Table 1). Furthermore, similar to B.1.1.7, Cypriot B.1 sequences clustered in the B.1.1.x (Figure 5) and B.basal clades (Figure S3), while B.1.177 sequences (Figure S4) did not all cluster together, as shown by the gray-shaded boxes (Figure 5, Figures S3 and S4). However, Cypriot B.1.2 sequences in the B.1.2 clade mostly clustered together (Figure S5, gray-shaded boxes).

**Figure 8:**
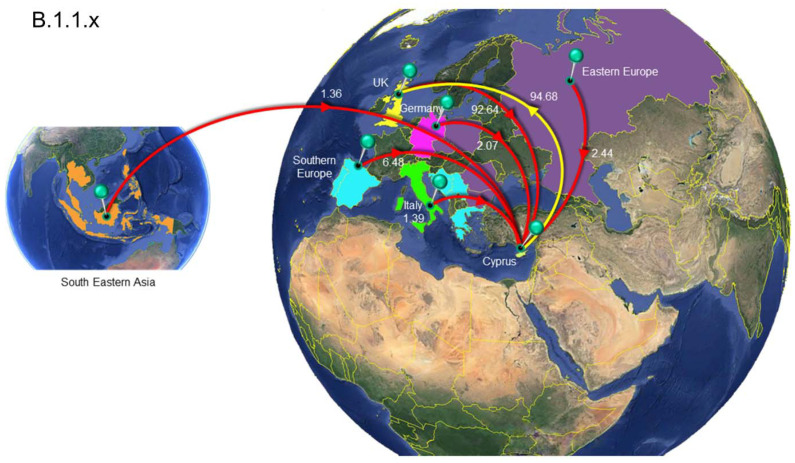
Map of SARS-CoV-2 B.1.1.x clade group transmission between Cyprus and other countries. The geographic origins of SARS-CoV-2 B.basal imported into Cyprus based on the statistical phylogeographic analysis are shown as red lines, and exports from Cyprus to other countries are shown as yellow lines. Countries and groups of countries acting as “sources” or “sinks” for SARS-CoV-2 B.1.1.x transmission are highlighted and labeled, and the average number of migration events is indicated. The highlighted area of Eastern Europe consists of Bulgaria, Romania, the Czech Republic, Poland and Russia, and the highlighted area of southern Europe consists of Bosnia and Herzegovina, Croatia, Greece, Montenegro, Serbia, Portugal and Spain. The highlighted area of southeastern Asia consists of Cambodia, the Philippines, Taiwan, Thailand, Indonesia, Singapore and Malaysia. Map images courtesy of Google Earth Pro 7.3.2.5776 (14 December 2015). Center: Global view centered on Europe. 36°16′38.78″ N 36°07′29.71″ E, Eye alt 7949.12 km. US Dept. of State Geographer, DATA SIO, NOAA, U.S. Navy, NGA, GEBCO. Image Landsat/Copernicus. 2018 © Google. Left: Southeastern Asia Region: 6°16′52.33″ N 113°55′29.68″ E -18 m, Eye alt 8880.55 km, Image Landsat/Copernicus, US Dept. of State Geographer, Data SIO, NOAA, U.S. Navy, NGA, GEBCO, © 2021 Google. https://www.google.com/earth/versions/#earth-pro (accessed on 10 April 2019 and 23 March 2021).

**Figure 9:**
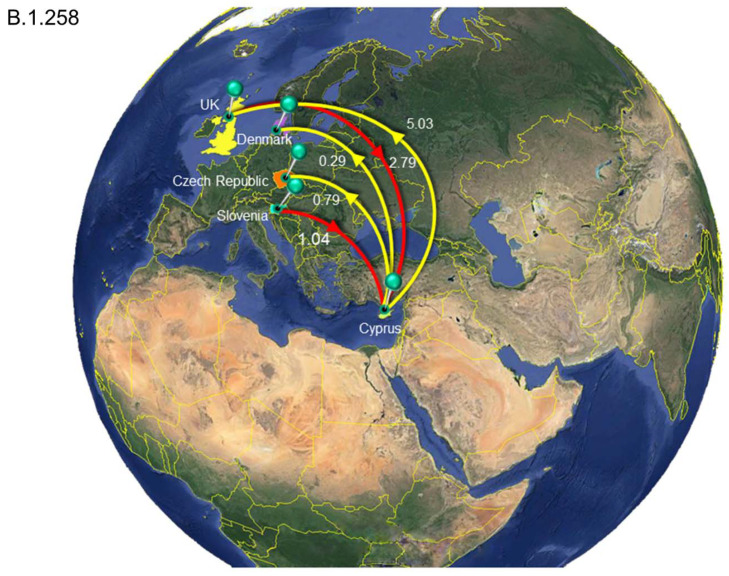
Map of SARS-CoV-2 B.1.258 clade group transmission between Cyprus and other countries. The geographic origins of SARS-CoV-2 B.1.258 imported into Cyprus based on the statistical phylogeographic analysis are shown as red lines, and exports from Cyprus to other countries are shown as yellow lines. Countries acting as “sources” or “sinks” for SARS-CoV-2 B.basal transmission are highlighted and labeled, and the average number of migration events is indicated. Map images courtesy of Google Earth Pro 7.3.2.5776 (14 December 2015). Global view centered on Europe. 36°16′38.78″ N 36°07′29.71″ E, Eye alt 7949.12 km. US Dept. of State Geographer, DATA SIO, NOAA, U.S. Navy, NGA, GEBCO. Image Landsat/Copernicus. 2018 © Google. https://www.google.com/earth/versions/#earth-pro (accessed on 10 April 2019).

**Figure 10:**
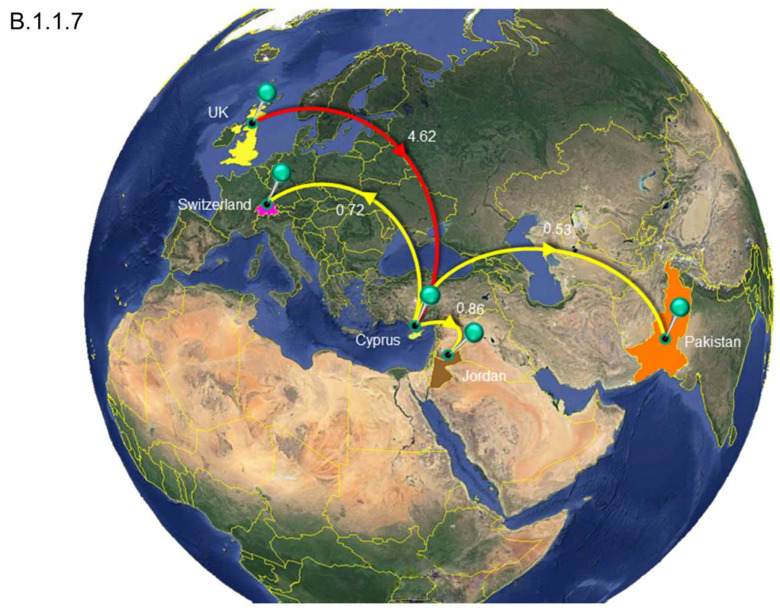
Map of SARS-CoV-2 B.1.1.7 clade group transmission between Cyprus and other countries. The geographic origins SARS-CoV-2 B.basal infection imported into Cyprus based on the statistical phylogeographic analysis are shown as red lines, and exports from Cyprus to other countries are shown as yellow lines. Countries and continents acting as “sources” or “sinks” for SARS-CoV-2 B.1.1.7 transmission are highlighted and labeled, and the average number of migration events is indicated. Map images courtesy of Google Earth Pro 7.3.2.5776 (14 December 2015). Global view centered on Europe. 36°16′38.78″ N 36°07′29.71″ E, Eye alt 7949.12 km. US Dept. of State Geographer, DATA SIO, NOAA, U.S. Navy, NGA, GEBCO. Image Landsat/Copernicus. 2018 © Google. https://www.google.com/earth/versions/#earth-pro (accessed on 10 April 2019).

The analysis to estimate importation into Cyprus led to the identification of 149 putative introduction events (95% HPD: 48–243) across the six datasets, with B.1.1.x accounting for the vast majority of these (~72%, Table 2). The UK was by far the most important source location, accounting for ~87% of all introductions into Cyprus (Table 2 and Table S4, Figure 8, Figure 9 and Figure 10, Figures S6–S8). In addition to the UK, which accounted for the majority of imports into Cyprus (~87%), the imports for the B.1.1.x clade originated from multiple countries/subregions, including eastern Europe (~2%), Germany (~2%), Italy (~1%), southeastern Asia (~1%), and southern Europe (~6%) (Table 2, Figure 8). The B.basal clade was also mostly imported from the UK (~89%) and to a lesser extent from Germany (~11%) (Table 2, Figure S6). The B.1.2 clade was the only clade without imports from the UK, with all imports being from the USA (Table 2, Figure S7). Similar to the B.1.1.x and B.basal clades, the B.1.258 clade was also mostly imported from the UK (~73%) (Table 2, Figure 9), with a smaller percentage of imports being from Slovenia (~27%), while for the B.1.177 and B.1.1.7 clades, all imports were from the UK (Table 2, Figure S8 and Figure 10).

A similar pattern was observed for SARS-CoV-2 exports from Cyprus to elsewhere; a total of ~77 (95% HPD: 17-132) export events were inferred, and ~90% of these were to the UK (Table 2 and Table S4). However, only two clades contained exports to the UK: the B.1.1.x clade, with all exports being to the UK (Table 2, Figure 8), and the B.1.258 clade (~82%) (Table 2, Figure 9). The other countries to which the B.1.258 clade was exported were the Czech Republic (~13%) and Denmark (~5%) (Table 2, Figure 9). The exports in clades B.basal, B.1.2, B.1.177 and B.1.1.7 were not to the UK. Specifically, for the B.basal clade, the majority of exports were to Chile (~76%) and Brazil (~23%) (Table 2, Figure S6). Clades B.1.2 and B.1.177 were exported to the USA and Finland, respectively (Table 2, Figures S7 and S8). Interestingly, although the B.1.1.7 clade contained the majority of imports from the UK, the exports were to Jordan (~41%), Pakistan (~25%) and Switzerland (~34%) (Table 2, Figure 10).

**Figure 11:**
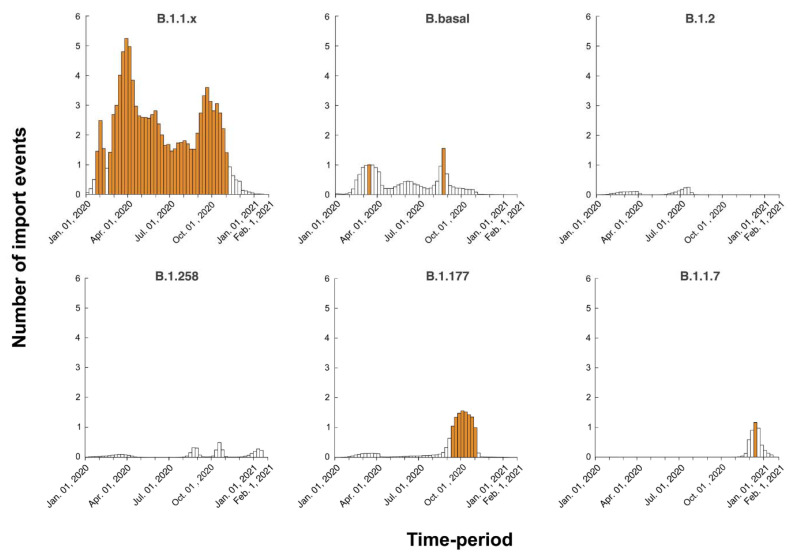
Temporal dynamics of SARS-CoV-2 introduction into Cyprus. The width of each bar corresponds to one week, and the height corresponds to the number of times that a migration event into Cyprus was inferred to have occurred in that week, averaged over the post-burn-in states sampled during the Markov Chain Monte Carlo (MCMC) integration. Weeks during which more than one import event was inferred are colored orange. The *y*-axis represents the number of import events per week, while the *x*-axis represents the sampling time-period from January 2020 to January 2021.

It is important to note that the degree of uncertainty in the estimated numbers of introduction and exportation events is large. Moreover, zero was not included in the 95% HPD interval for only a few migration links (Table 2). The expected times of migration events into Cyprus were estimated through Markov jumps, and the temporal dynamics were summarized for each dataset (Figure 11). As these estimated timings are averaged over the entire set of postburn-in trees sampled during Markov chain Monte Carlo (MCMC) integration, and uncertainty in the migration event timings can be considerable, there is often less than one migration event per week. However, for B.1.1.x, we inferred that there was at least one import event every week on average, with a peak of five events between 19 February 2020, and 29 October 2020. Similarly, for B.1.177, there was a period of seven consecutive weeks between 10 September 2020, and 29 October 2020, with multiple import events.

**Figure 12:**
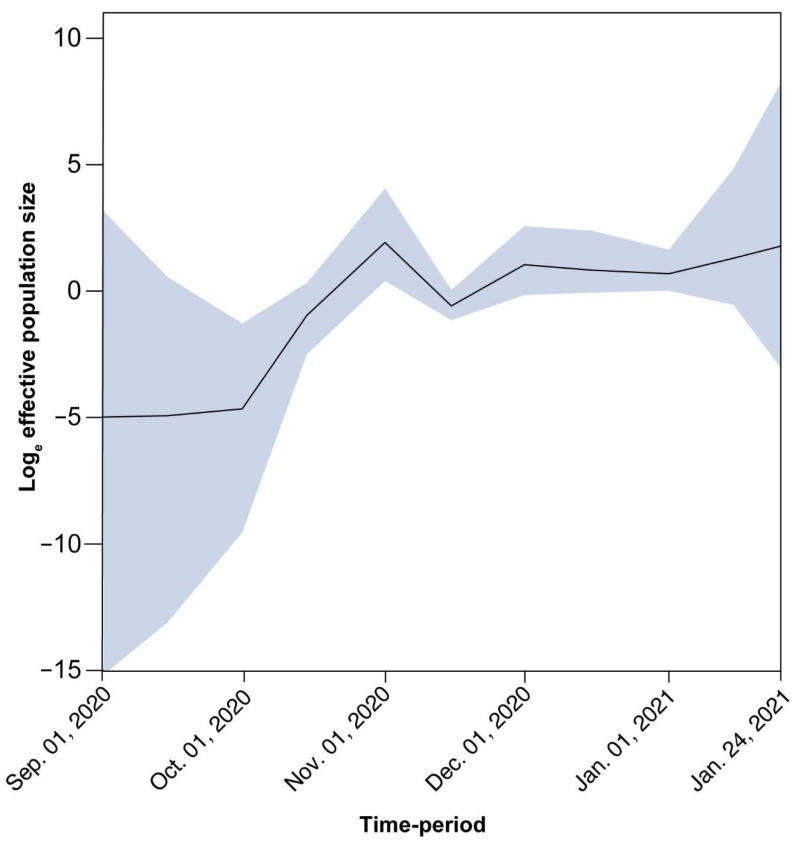
Population size changes in the large Cyprus-specific B.1.258 transmission lineage. The solid black line represents the estimated mean of the effective population size on a log_e_ scale (represented by the *y*-axis) through the sampling period from September 2020 to January 2021 (represented by the *x*-axis). The effective population size can be thought of as the number of individuals who contribute offspring to the descending generation. The blue-shaded area marks the associated 95% HPD interval.

For B.1.258, there is a large and nearly perfectly supported clade (Figure 6 gray-shaded box and Figure 12) (posterior support = 0.99) that consists almost exclusively of genomes sampled from Cyprus. Of the 240 taxa, only nine are from the UK and two are from Denmark. This B.1.258 clade represents a highly successful Cypriot transmission lineage, of which the demographic dynamics were reconstructed in the same way as for the complete dataset after discarding the non-Cypriot taxa. The spread of this lineage started on approximately 23 September 2020 (95% HPD: 3 September 2020–1 October 2020) with an initial period of exponential growth until November 2020, after which a rather stable plateau was reached which persisted through the most recent sampling date. For the other clades, we did not find such a successful Cyprus-specific transmission lineage (Figure 5, Figure 6 and Figure 7, Figures S3–S5). Specifically, the largest well-supported (i.e., posterior support ≥ 0.9) clades with at least 90% of taxa from Cyprus contained 15 (B.1.177), 7 (B.1.2), 4 (B.1.1.7), 5 (B.1.1x) and 6 (B.basal) taxa (Figure 5 and Figure 7, Figures S3–S5). 

## 4. Discussion

In the current study, 596 nearly complete SARS-CoV-2 genome sequences were analyzed, and 34 SARS-CoV-2 lineages were detected from three distinct cohorts in Cyprus during the period of April 2020 to January 2021. The most prevalent lineages in this time-period were B.1.258, B.1.1.29, B.1.177, B.1.2, B.1 and B.1.1.7.

In chronological order of presence and prevalence, the B.1.1.29 lineage was found mostly in April 2020 at the start of the sampling period, with few samples detected in May and even fewer in June (Table 1 and Figure 1). The timed migration analysis estimated that the date of introduction of the B.1.1.x clade (mostly comprising lineage B.1.1.29) to the island was 20 January 2020 (B.1.1.x, 95% HPD: 2 January 2020–5 February 2020), which may imply that this clade had cryptic characteristics from the time of import until sampling. Notably, the reason why the B.1.1.x clade is not composed of only B.1.1.29 is that some genomes in this study were not complete, and gap regions that encompass lineage-defining mutations are used by Pangolin [36]. Furthermore, sequences of the B.1 lineage (the second most prevalent lineage during that period) also partially fell within the B.1.1.x clade (*n* = 13), and the remaining B.1 sequences fell within the B.basal clade (*n* = 5). Interestingly, however, exports of the B.basal clade were to Latin America (Figure S6), while exports of the B.1.1.x clade were mostly to the UK (Figure 8).

In other studies, lineage B.1.1.29 was reported to have been commonly identified in Europe, particularly the UK [36,64]; this is supported by the findings of our study, in which the majority of the migration events of the B.1.1.x clade originated from the UK (Table 2, Figure 8). Interestingly, this lineage was also reported in March–April of 2020 in Cape Town, South Africa, and it was estimated to have been imported from the Netherlands [64]. However, the B.1.1.29 lineage was identified much less frequently in Cyprus after June 2020. Considering the timeline of the prevalence of this lineage, it is speculated that the travel bans and inland restrictions at the start of the epidemic in Cyprus, along with the measures established by the Cypriot government at the end of March and beginning of April [65], were able to slow the spread of this lineage. The B.1 lineage, on the other hand, is a large European lineage for which the origin roughly aligns with the northern Italy outbreak early in 2020 (24 January 2020) [36]. Similar to B.1.1.29, the majority of this lineage was also observed in only the beginning of the epidemic in Cyprus, suggesting that this lineage was also affected by the measures established by the Cypriot government during that period.

It is important to note that the only common mutation found in B.1.1.29 and the B.1 lineage was D614G (Figure 2). This mutation quickly became dominant in viral strains by April 2020, as it conferred various benefits upon SARS-CoV-2. This mutation in the S protein promotes enhanced binding to human angiotensin-converting enzyme 2 (ACE2), resulting in increased infectivity, replication, and transmission [22,66,67]. These benefits allow viral lineages carrying the D614G mutation to outcompete those lacking it during transmission bottlenecks, explaining its dominance and prevalence [67]. The sequences of the B.1.1.29 and B.1 lineages examined in this study also contained several uncommon mutations. Among the 18 B.1 sequences that were identified in this study, seven uncommon mutations were found, none of which were localized in the RBD, while among the 147 B.1.1.29 sequences, 21 uncommon mutations were found, seven of which were localized in the RBD (Table S3). Namely, the uncommon G446V mutation has been reported to reduce serum binding and neutralization [68]. The fact that such mutations can develop in a short amount of time highlights the risk of increased prevalence and establishment in future viral generations.

From July–September 2020 in Cyprus, no lineage was as prevalent as the B.1.1.29 lineage in April–June 2020. The July–September 2020 period was characterized by B.1.2, B.1.1.x and B.basal import events (Table 1 and Figure 11), yet there was no large-scale outbreak. Climate effects may play a role in constraining the transmission of the virus; however, the spread of the epidemic cannot be substantially limited by environmental factors alone [69]. Thus, the lower rate of infection in July–September 2020 was probably due to mitigation measures, such as limitations on social gatherings and travel restrictions [65]. The travel restrictions were partially lifted on 20 June 2020, for countries listed in categories A and B, from which travel was allowed as long as passengers showed their certificate indicating a negative SARS-CoV-2 PCR result [65]. The travel categories were based on epidemiological status and the health and safety measures implemented within the countries in each category. Category A consisted of Greece, Malta, Bulgaria, Norway, Austria, Finland, Slovenia, Hungary, Israel, Denmark, Germany, Slovakia, and Lithuania, and Category B consisted of Switzerland, Poland, Romania, Croatia, Estonia, and the Czech Republic [65,70].

An in-depth look at the sequences in the B.1.2 lineage, which was the most prevalent lineage in the summer months, revealed that this lineage was most commonly reported in the USA, with the earliest date of identification being 2 February 2020 [36,71]. The B.1.2 lineages in this study also clustered together with the USA samples (Table 2 and Figure S7). Furthermore, our data estimated that the date of import was 31 March 2020 (B.1.2, 95% HPD: 31 January 2020–22 July 2020). According to the actual collection dates of the samples in this lineage, one sequence was detected in April and one sequence was detected in July, while the majority of sequences were detected in August (*n* = 14), and a few were detected in September (*n* = 3). The time-period (April–August 2020) between the first introduction and identification of the majority of sequences may signify that the lineage was being transmitted without being detected, and there was an influx of new introductions (Figure 11).

The only common S protein mutation identified in the B.1.2 lineage in this study was D614G, while detected uncommon S protein mutations included G769V, A942V and S1170X (Tables S2 and S3). Since the functional analysis of SARS-CoV-2 mutations is currently under global scientific review, available data on these uncommon mutations are scarce. Nonetheless, specifically for mutations such as G769V, mutational frequency data prompted the suggestion they do not impede the evolutionary stability of the virus [72]. As such, they may not be selected against, and increased frequencies of these mutations may become apparent in the future.

The dynamic nature of the Cypriot epidemic is apparent from the alternating predominance of different lineages. The importation of the most prevalent lineage in Cyprus, B.1.258, was estimated to have occurred on March 7, 2020 (95% HPD: 17 January 2020–16 April 2020), since two B.1.258 samples were detected on the island during the months of April and May. It is possible that the measures in place during that period mitigated the spread of this lineage. The B.1.258 lineage was first identified globally on 22 March 2020, and it was most commonly detected in the UK [36]. After the prohibition of flight travel in the beginning of April (4 April 2020), the UK was finally included in category B on 1 August 2020, allowing travel to and from Cyprus under certain conditions (e.g., a negative PCR test) [65]; this may explain the import events identified, as shown in Figure 11. Additionally, most B.1.258 lineage migration events identified in this study were to and from the UK (Table 2, Figure 9).

The end of the summer 2020 period, specifically September 2020, marked the beginning of the increase in the B.1.258 lineage, which became the most prevalent lineage in Cyprus (Figure 1). Starting in September, specifically approximately 23 September 2020 (95% HPD: 3 September 2020–1 October 2020), B.1.258 continued to increase in prevalence every month, with exponential growth until November, when it stabilized until the end of the sampling period (Figure 12). This signifies that the measures established by the government (on 5 November 2020), such as the curfews and limitations/closing of catering establishments, due to the sudden increase in SARS-CoV-2 infection cases during the September–November 2020 period, had begun to have an effect [65].

The B.1.258 lineage was also prevalent in the Czech Republic and appeared in Denmark and the UK during the same period (September to December 2020) [73]. These three countries were identified as “sources” and “sinks” within our dataset for the B.1.258 lineage (Table 2, Figure 9). The B.1.258 lineage is characterized by ΔH69/V70 deletions in the S protein (Figure 2 and Figure 3), and interestingly, these deletions have recurrently emerged in a number of lineages, including B.1.1.7 and B.1.351 [73,74,75]. ΔH69/V70 deletions in the S protein (specifically the N-terminal domain (NTD)) have been linked to increased infectivity and evasion of the host immune system. These two deletions are suspected to compensate for mutations such as N501Y that reduce infectivity [73,74,75]. Moreover, ΔH69/V70 has been reported to be responsible for the failure of certain commercial testing kits to detect the S protein [62]. The other mutation commonly detected in the sequences in this lineage was N439K (Figure 2 and Figure 3). This mutation has been reported to have evolved independently multiple times [73]. N439K lies within the RBD, has been reported to be associated with immune escape, and confers increased binding affinity upon the ACE2 receptor, resulting in infection with a similar clinical outcome and a marginally higher viral load. An increase in viral load can increase the chance of transmission, though the chance is small [76]. In one sequence in the B.1.258 lineage in this study, the K417N mutation was detected (Figure 2B). The K417N amino acid substitution is part of a group of substitutions most frequently detected in South African lineage B.1.351 (N501Y, K417N and E484K) that possibly promote antibody evasion [77]. Interestingly, it has also been found that K417N weakens binding between the RBD and human ACE2, while other mutations, such as E484K, enhance binding [77]. The independent evolution of certain mutations and deletions (ΔH69/V70, N439K and K417N) in different lineages highlights the importance of molecular epidemiology studies, in which known and dangerous mutations can be identified and monitored.

During the last four months of sampling, in addition to the most prevalent lineage, B.1.258, two other lineages were notable, namely, B.1.177 and B.1.1.7. Chronologically, in this study, the first B.1.177 sample was detected on 12 March 2020 (B.1.177, 95% HPD: 2 February 2020–8 April 2020). Even though the first sample in Cyprus was identified in April 2020, the majority of the B.1.177 samples were detected from October 2020–January 2021. This lineage was predominantly detected in the UK and has since circulated around Europe [36], with detection in countries such as France, Ireland, Austria, Belgium, Finland, the Netherlands and Norway and even Ontario, Canada [78,79]. The data analysis in the present study indicated that this lineage was imported to Cyprus from the UK and exported to Finland (Table 2, Figure S8).

In addition to D614G, the most common mutations identified in the B.1.177 lineage in this study were L18F and A222V (Figure 2). Furthermore, one uncommon mutation, S477G, was identified in the RBD. The L18F substitution, also found in the South African strain B.1.351, has been reported to confer antibody escape [80], and it has been speculated that A222V may contribute to spreading efficiency as well as immune evasion; however, this is still under investigation [81]. The uncommon mutation S477G, on the other hand, has been reported to strengthen the interaction between ACE2 and the RBD [82], thereby highlighting the dangers of new evolutionary traits, even in lineages that do not normally harbor such mutations.

The B.1.1.7 lineage was identified in late September 2020 and immediately started outcompeting other SARS-CoV-2 lineages in the UK [62,63]. In Cyprus, the lineage was estimated to have arrived on 1 December 2020 (B.1.1.7, 95% HPD: 8 November 2020–19 December 2020), and the first sample was detected at the end of December. The results of this study showed that this lineage was indeed imported to Cyprus from the UK, and it was exported to Switzerland, Jordan and Pakistan (Table 2, Figure 10). The B.1.1.7 lineage is extremely concerning since it is rapidly spreading around the globe and has already been detected in 82 countries, indicating that the transmission rate is as high as 59–74% [62].

The B.1.1.7 lineage is considered to pose a high potential threat due to the mutations/deletions it harbors. The following mutations within the S protein have been reported in this lineage: N501Y, A570D, P681H, T716I, S982A, D1118H, ΔH69/V70 and ΔY144 [36]. These mutations, which were also detected in our study (Figure 2), have been reported to confer resistance upon antibody neutralization, increase transmissibility, and possibly induce greater disease severity [62]. Furthermore, the S98F (50%, 5/10) and S162G (40%, 4/10) substitutions were also found in our dataset, with relatively high frequencies, highlighting the possibility of the further accumulation of mutations (Table S2).

In this study, one vivid result from the phylogeographic analysis is the finding that the majority of imports of SARS-CoV-2 lineages in Cyprus were from the UK, with other regions and countries identified as sources of SARS-CoV-2 import to a lesser extent (Figure 8, Figure 9 and Figure 10). Specifically, the fact that the majority of imports of SARS-CoV-2 for the most prevalent lineages were from the UK, especially from B.1.1.29 (in clade B.1.1.x), B.1.258 as well as B.1.1.7, which represent the three consecutive waves of lineages in Cyprus (Figure 1), is of significant importance. These findings represent a clear course of action for the public health control of SARS-CoV-2, through travel restrictions and management strategies towards major geolocation sources of SARS-CoV-2 lineages, and thus safeguarding public health.

In conclusion, this molecular epidemiological study showed the dynamicity of the epidemic from April 2020 to January 2020 in Cyprus, during which a total of 34 lineages were identified, with different lineages being prevalent at different time points. By examining the temporal and spatial characteristics of the epidemic, it was shown that the highest number of infections occurred after the summer 2020 period, when the measures in place and the travel restrictions were lifted, and that the UK played a major role in SARS-CoV-2 migration events in Cyprus. These results not only provide insights for evaluating the efficacy of and improving public health measures, but also reveal the lineages and, to an extent, the common and uncommon mutations/deletions identified in Cyprus. This information is very important for monitoring the evolution of the virus and evaluating the efficacy of diagnostic kits, therapeutic drugs and vaccines, both in Cyprus and abroad.

## Figures and Tables

**Table 1:** SARS-CoV-2 lineages identified from 596 samples in Cyprus from April 2020 to January 2021.

Time-Period	April–June 2020	July–September 2020	October–November 2020	December 2020–January 2021	Total
Lineage	Number of Sequences Per Lineage (%)	Number of Sequences Per Lineage (%)	Number of Sequences Per Lineage (%)	Number of Sequences Per Lineage (%)	Number of Sequences Per Lineage (%)
A	1 (0.51)	-	-	-	1 (0.17)
B	5 (2.56)	-	-	-	5 (0.84)
B.1	17 (8.72)	1 (1.32)	-	-	18 (3.02)
B.1.1.1	-	6 (7.89)	1 (0.61)	-	7 (1.17)
B.1.1.7	-	-	-	10 (6.17)	10 (1.68)
B.1.1.29	144 (73.85)	-	1 (0.61)	2 (1.23)	147 (24.66)
B.1.1.41	4 (2.05)	-	-	-	4 (0.67)
B.1.1.67	-	1 (1.32)	-	-	1 (0.17)
B.1.1.130	1 (0.51)	-	-	-	1 (0.17)
B.1.1.131	2 (1.03)	-	-	-	2 (0.34)
B.1.1.141	-	-	-	2 (1.23)	2 (0.34)
B.1.1.153	-	-	3 (1.84)	-	3 (0.5)
B.1.1.159	1 (0.51)	-	1 (0.61)	-	2 (0.34)
B.1.1.161	5 (2.56)	-	-	-	5 (0.84)
B.1.1.192	-	2 (2.63)	-	-	2 (0.34)
B.1.1.218	-	1 (1.32)	1 (0.61)	-	2 (0.34)
B.1.1.230	-	1 (1.32)	-	-	1 (0.17)
B.1.1.251	4 (2.05)	-	-	-	4 (0.67)
B.1.1.277	4 (2.05)	-	-	-	4 (0.67)
B.1.1.288	-	2 (2.63)	-	-	2 (0.34)
B.1.1.307	-	1 (1.32)	-	-	1 (0.17)
B.1.1.315	-	-	-	1 (0.62)	1 (0.17)
B.1.1.317	-	2 (2.63)	-	-	2 (0.34)
B.1.2	1 (0.51)	18 (23.68)	-	-	19 (3.19)
B.1.36	-	2 (2.63)	-	-	2 (0.34)
B.1.160	-	1 (1.32)	1 (0.61)	-	2 (0.34)
B.1.177	1 (0.51)	-	30 (18.4)	10 (6.17)	41 (6.88)
B.1.177.8	-	-	1 (0.61)	-	1 (0.17)
B.1.221.1	-	-	1 (0.61)	-	1 (0.17)
B.1.236	-	6 (7.89)	-	-	6 (1.01)
B.1.258	2 (1.03)	32 (42.11)	123 (75.46)	136 (83.95)	293 (49.16)
B.1.258.17	-	-	-	1 (0.62)	1 (0.17)
B.1.313	2 (1.03)	-	-	-	2 (0.34)
B.6	1 (0.51)	-	-	-	1 (0.17)
Total	195	76	163	162	596

**Table 2:** The estimated number of migration events towards and from Cyprus. Lower and upper refer to the bounds of the 95% HPD interval.

Clade.Lineage ^1^	From ^2^	To ^3^	Average ^4^	Lower ^5^	Upper ^6^
**Clade.B.1.1.x**	All	Cyprus	106.38	34	161
	Eastern Europe	Cyprus	2.44	0	8
	Germany	Cyprus	2.07	0	7
	Italy	Cyprus	1.39	0	5
	South Eastern Asia	Cyprus	1.36	0	5
	Southern Europe	Cyprus	6.48	0	13
	United Kingdom	Cyprus	92.64	27	142
	Cyprus	All	64.68	14	104
	Cyprus	United Kingdom	64.68	14	104
**Clade.B.basal**	All ^7^	Cyprus	17.75	8	25
	Germany	Cyprus	1.95	0	6
	United Kingdom	Cyprus	15.8	6	24
	Cyprus	All	1.44	1	3
	Cyprus	Brazil	0.33	0	1
	Cyprus	Chile	1.1	0	2
**Clade.B.1.2**	All	Cyprus	1.94	1	3
	USA	Cyprus	1.94	1	3
	Cyprus	All	1.74	1	5
	Cyprus	USA	1.74	1	5
**Clade.B.1.258**	All	Cyprus	3.84	1	5
	Slovenia	Cyprus	1.04	0	2
	United Kingdom	Cyprus	2.79	0	4
	Cyprus	All	6.11	2	10
	Cyprus	Czech Republic	0.79	0	3
	Cyprus	Denmark	0.29	0	2
	Cyprus	United Kingdom	5.03	1	9
**Clade.B.1.177**	All	Cyprus	13.99	11	17
	United Kingdom	Cyprus	13.99	11	17
	Cyprus	All	1.01	1	1
	Cyprus	Finland	1.01	1	1
**Clade.B.1.1.7**	All	Cyprus	4.62	3	7
	United Kingdom	Cyprus	4.62	3	7
	Cyprus	All	2.11	1	4
	Cyprus	Jordan	0.86	0	2
	Cyprus	Pakistan	0.53	0	1
	Cyprus	Switzerland	0.72	0	2

^1^ The name of each Clade.Lineage represented by a singular lineage except for B.basal and B.1.1.x. The lineages contained by B.basal and B.1.1.x are available in the results section “The phylogenetic analysis of Cypriot SARS-CoV-2 infected individuals” (first paragraph). ^2^ “From” indicates the migration events of a country/subregion from which migration events were initiated from. Countries/subregions are as denoted by United Nations geographical subregion. ^3^ “To” indicates the migration events from a country/subregion from which migration events were directed to. Countries/subregions are as denoted by United Nations geographical subregion. ^4–6^ Represent average Markov jumps based on the lower and upper bounds of the of the 95% HPD interval migration events towards and from Cyprus. ^7^ “All” Represents the aggregation of the migration events from each country/subregion.

## Data Availability

The sequences will be available after acceptance on the GISAID database (https://www.gisaid.org/, accessed on 15 February 2021). The results will be available upon request from the corresponding author.

## Supplementary Materials

The following are available online at https://www.mdpi.com/article/10.3390/v13061098/s1, Figure S1: Root-to-tip divergence as a function of sampling time for the different datasets, Figure S2: Histogram of the time to the most recent common ancestor (tMRCA) estimates of the B.1.258 clade, Figure S3: Time-scaled migration history for the B.basal dataset, Figure S4: Time-scaled migration history for the B.1.177 dataset, Figure S5: Time-scaled migration history for the B.1.2 dataset, Figure S6: Map of SARS-CoV-2 B.basal clade group transmission between Cyprus and other countries, Figure S7: Map of SARS-CoV-2 B.1.2 clade group transmission between Cyprus and other countries shows imports from and exports to only the United States, Figure S8: Map of SARS-CoV-2 B.1.177 clade group transmission between Cyprus and other countries. Table S1: The number of genomes for each country in the B.1.1.x data set, Table S2: Common mutations/deletions identified in the S proteins of the most prevalent lineages (B.1.258, B.1.1.29, B.1.177, B.1.2, B.1 and B.1.1.7) in Cyprus, Table S3: Uncommon mutations/deletions identified in the S proteins of the most prevalent lineages (B.1.258, B.1.1.29, B.1.177, B.1.2, B.1 and B.1.1.7) in Cyprus, Table S4: The estimated number of migration events towards and from Cyprus for each dataset. Lower and upper refer to the bounds of the 95% HPD interval.
