# Supplementary material for: A Comprehensive Molecular Epidemiological Analysis of SARS-CoV-2 Infection in Cyprus from April 2020 to January 2021: Evidence of a Highly Polyphyletic and Evolving Epidemic
Source: Viruses. 2021 Jun 9;13(6):1098. doi: 10.3390/v13061098 (PMC8227210; doi:10.3390/v13061098)
Supplement: Supplementary file 1 [file viruses-13-01098-s001.zip › viruses-1231469-supplementary.pdf]

**Table S1.** The number of genomes for each country in the B.1.1.x data set.

| Number of Countries | Country | Taxon[1] Count |
|---|---|---|
| 1 | Bangladesh | 1 |
| 2 | Bosnia and Herzegovina | 1 |
| 3 | Bulgaria | 1 |
| 4 | Cambodia | 1 |
| 5 | Chile | 1 |
| 6 | Colombia | 1 |
| 7 | Croatia | 1 |
| 8 | Guadeloupe | 1 |
| 9 | Guam | 1 |
| 10 | Latvia | 1 |
| 11 | Lithuania | 1 |
| 12 | Madagascar | 1 |
| 13 | Morocco | 1 |
| 14 | Papua New Guinea | 1 |
| 15 | Philippines | 1 |
| 16 | Romania | 1 |
| 17 | Saint Martin | 1 |
| 18 | Taiwan | 1 |
| 19 | Uganda | 1 |
| 20 | Uruguay | 1 |
| 21 | Yemen | 1 |
| 22 | Nigeria | 2 |
| 23 | Sierra Leone | 2 |
| 24 | Tunisia | 2 |
| 25 | Zambia | 2 |
| 26 | China | 3 |
| 27 | Greece | 3 |
| 28 | Montenegro | 3 |
| 29 | Serbia | 3 |
| 30 | South Africa | 3 |
| 31 | Thailand | 3 |
| 32 | Iceland | 4 |
| 33 | Indonesia | 4 |
| 34 | Ireland | 4 |
| 35 | Peru | 4 |
| 36 | Portugal | 4 |
| 37 | Austria | 5 |
| 38 | Czech Republic | 5 |
| 39 | Singapore | 5 |
| 40 | Turkey | 5 |
| 41 | Finland | 8 |
| 42 | Mexico | 8 |
| 43 | Poland | 8 |
| 44 | Israel | 9 |
| 45 | Russia | 9 |

| | | |
|---|---|---|
| 46 | United Arab Emirates | 9 |
| 47 | Brazil | 11 |
| 48 | Netherlands | 11 |
| 49 | Argentina | 12 |
| 50 | Belgium | 12 |
| 51 | Japan | 12 |
| 52 | Malaysia | 12 |
| 53 | Norway | 12 |
| 54 | Saudi Arabia | 12 |
| 55 | South Korea | 15 |
| 56 | Spain | 17 |
| 57 | France | 18 |
| 58 | Australia | 20 |
| 59 | Sweden | 22 |
| 60 | Denmark | 23 |
| 61 | India | 23 |
| 62 | Italy | 24 |
| 63 | Switzerland | 30 |
| 64 | Germany | 40 |
| 65 | Canada | 87 |
| 66 | United Kingdom | 166 |
| 67 | Cyprus | 200 |
| 68 | USA | 229 |

[1] Indicates the number of genomes that were identified per country in the B.1.1.x data set.

**Table S2.** Common mutations/deletions identified in the S proteins of the most prevalent lineages (B.1.258, B.1.1.29, B.1.177, B.1.2, B.1 and B.1.1.7) in Cyprus.

| Common Mutations | Number of sequences identified | Uncommon Deletions | Number of sequences identified | Total number of sequences of lineage |
|---|---|---|---|---|
| | | **Lineage** | | |
| | | **B.1** | | |
| D614G | 17 | -[1] | - | 18 |
| | | **B.1.1.7** | | |
| S98F*[2] | 5 | ΔH69 | 10 | |
| S162G* | 4 | ΔV70 | 10 | |
| N501Y | 10 | ΔY144 | 10 | |
| A570D | 10 | - | - | |
| D614G | 10 | - | - | 10 |
| P681H | 10 | - | - | |
| T716I | 10 | - | - | |
| S982A | 10 | - | - | |
| D1118H | 10 | - | - | |
| | | **B.1.1.29** | | |
| D614G | 147 | - | - | 147 |
| | | **B.1.177** | | |
| L18F | 33 | - | - | |
| A222V | 39 | - | - | 41 |
| D614G | 41 | - | - | |
| | | **B.1.2** | | |
| D614G | 19 | - | - | 19 |
| | | **B.1.258** | | |
| N439K | 291 | ΔH69 | 290 | 293 |
| D614G | 293 | ΔV70 | 290 | |

[1] Dashes indicate that there were no applicable data be input in a cell of the table

[2] Asterisks represent mutations/deletions that were found in approximately half of the sequences of the B.1.1.7 lineage; S98F found in 5/10 sequences and S162G found in 4/10.

**Table S3.** Uncommon mutations/deletions identified in the S proteins of the most prevalent lineages (B.1.258, B.1.1.29, B.1.177, B.1.2, B.1 and B.1.1.7) in Cyprus.

| Uncommon Mutations | Number of sequences identified | Uncommon Deletions | Number of sequences identified | Total number of sequences of lineage |
|---|---|---|---|---|
| | | **Lineage** | | |
| | | **B.1** | | |
| H49Y | 1 | -[1] | - | 18 |
| L176I | 1 | - | - | |
| F238L | 1 | - | - | |
| E654A | 1 | - | - | |
| E661Q | 1 | - | - | |
| M1029V | 1 | - | - | |
| M1237I | 1 | - | - | |
| | | **B.1.1.7** | | |
| - | - | - | - | 10 |
| | | **B.1.1.29** | | |
| C15Y | 1 | ΔQ14 | 1 | 147 |
| T124N | 1 | ΔC15 | 1 | |
| F338X | 1 | ΔV16 | 1 | |
| F342X | 1 | ΔN17 | 1 | |
| A344P | 1 | ΔL18 | 1 | |
| V367L | 1 | ΔT19 | 1 | |
| G446V | 1 | ΔN125 | 1 | |
| P507L | 1 | - | - | |
| V510L | 1 | - | - | |
| Q675H | 1 | - | - | |
| R683Q | 2 | - | - | |
| I805X | 1 | - | - | |
| A846V | 2 | - | - | |
| S937L | 1 | - | - | |
| V987L | 1 | - | - | |
| F1075L | 1 | - | - | |
| H1083Y | 1 | - | - | |
| R1091C | 1 | - | - | |
| L1203I | 1 | - | - | |
| G1251V | 1 | - | - | |
| S1252C | 1 | - | - | |
| | | **B.1.177** | | |
| S98F | 7 | - | - | 41 |
| M177I | 2 | - | - | |
| A262S | 1 | - | - | |
| P272L | 1 | - | - | |
| S477G | 1 | - | - | |
| V642F | 1 | - | - | |

| | | | | |
|---|---|---|---|---|
| E654V | 1 | - | - | |
| T859I | 1 | - | - | |
| A1174V | 1 | - | - | |
| M1237I | 1 | - | - | |
| | | **B.1.2** | | |
| G769V | 3 | - | - | |
| A942V | 1 | - | - | 19 |
| S1170X | 1 | - | - | |
| | | **B.1.258** | | |
| T20I | 2 | - | - | |
| Y28H | 2 | - | - | |
| W152L | 1 | - | - | |
| L176F | 1 | - | - | |
| I233V | 2 | - | - | |
| A243V | 6 | - | - | |
| G257V | 2 | - | - | |
| E281K | 1 | - | - | |
| T299I | 1 | - | - | |
| F374I | 1 | - | - | |
| K417N | 1 | - | - | |
| K528E | 1 | - | - | |
| T581S | 1 | - | - | |
| Q613H | 1 | - | - | |
| T618I | 1 | - | - | 293 |
| E654Q | 3 | - | - | |
| I666X | 1 | - | - | |
| Q675L | 2 | - | - | |
| Q677H | 1 | - | - | |
| M731I | 1 | - | - | |
| P793S | 2 | - | - | |
| T859I | 1 | - | - | |
| A893S | 3 | - | - | |
| S968A | 3 | - | - | |
| V1104L | 1 | - | - | |
| E1151D | 1 | - | - | |
| D1163Y | 10 | - | - | |
| V1176F | 2 | - | - | |
| L1203F | 2 | - | - | |

[1] Dashes indicate that there were no applicable data be input in a cell of the table

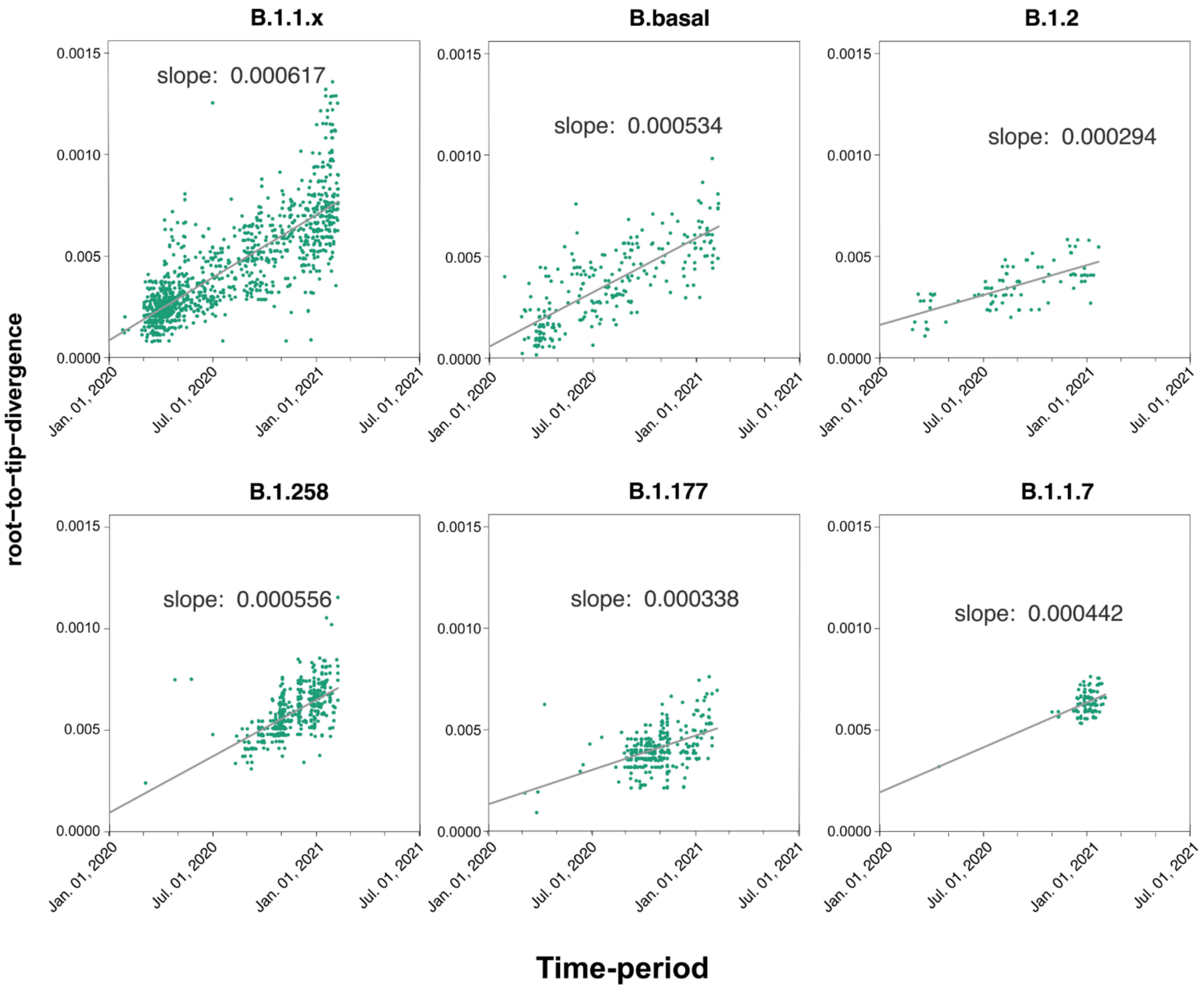


**Figure S1. Root-to-tip divergence as a function of sampling time for the different datasets.** Trees were rooted according to the heuristic residual mean squared criterion using TempEst [1]. Each green circle corresponds to a sequence in the dataset. The y-axis represents the root-to-tip genetic distance (in units of substitutions per site). The x-axis represents the sampling time from January 2020 to January 2021. The gray line corresponds to the best fit when regressing sampling time versus root-to-tip divergence, and the slope indicates the corresponding evolutionary rate estimate. For B.1.1.x, three sequences for which the sampling date was specified only in the year 2021 were omitted.

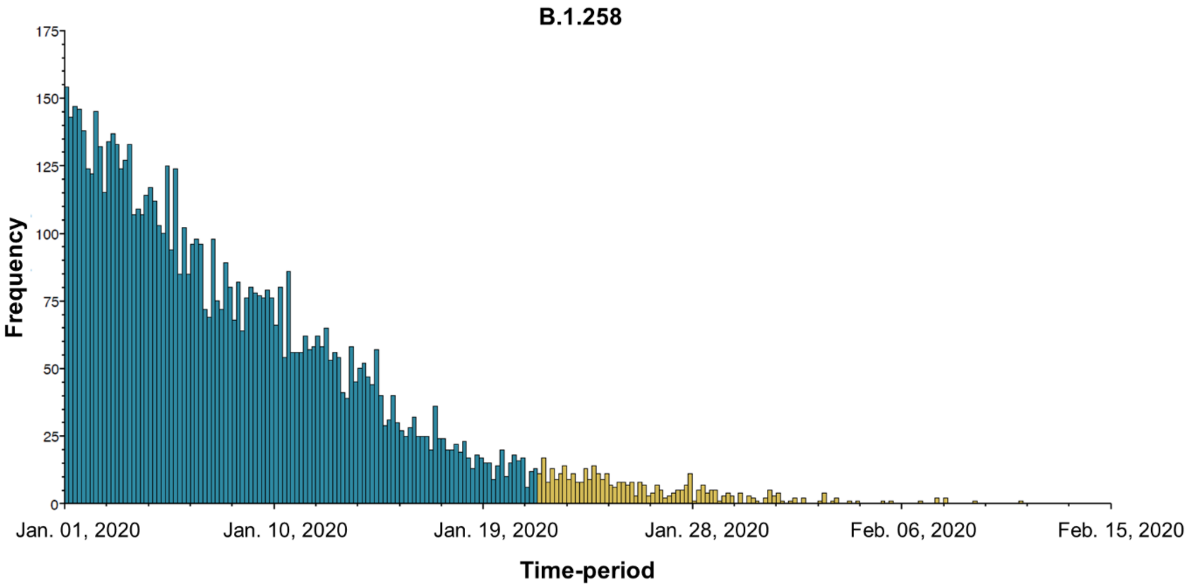


**Figure S2. Histogram of the time to the most recent common ancestor (tMRCA) estimates of the B.1.258 clade.** The y-axis represents the estimated tMRCA frequency in the B.1.258 lineage at the corresponding moment in time, which is indicated by the x-axis. Blue-colored bars indicate the 95% HPD interval, while yellow bars indicate 5% of the other samples.

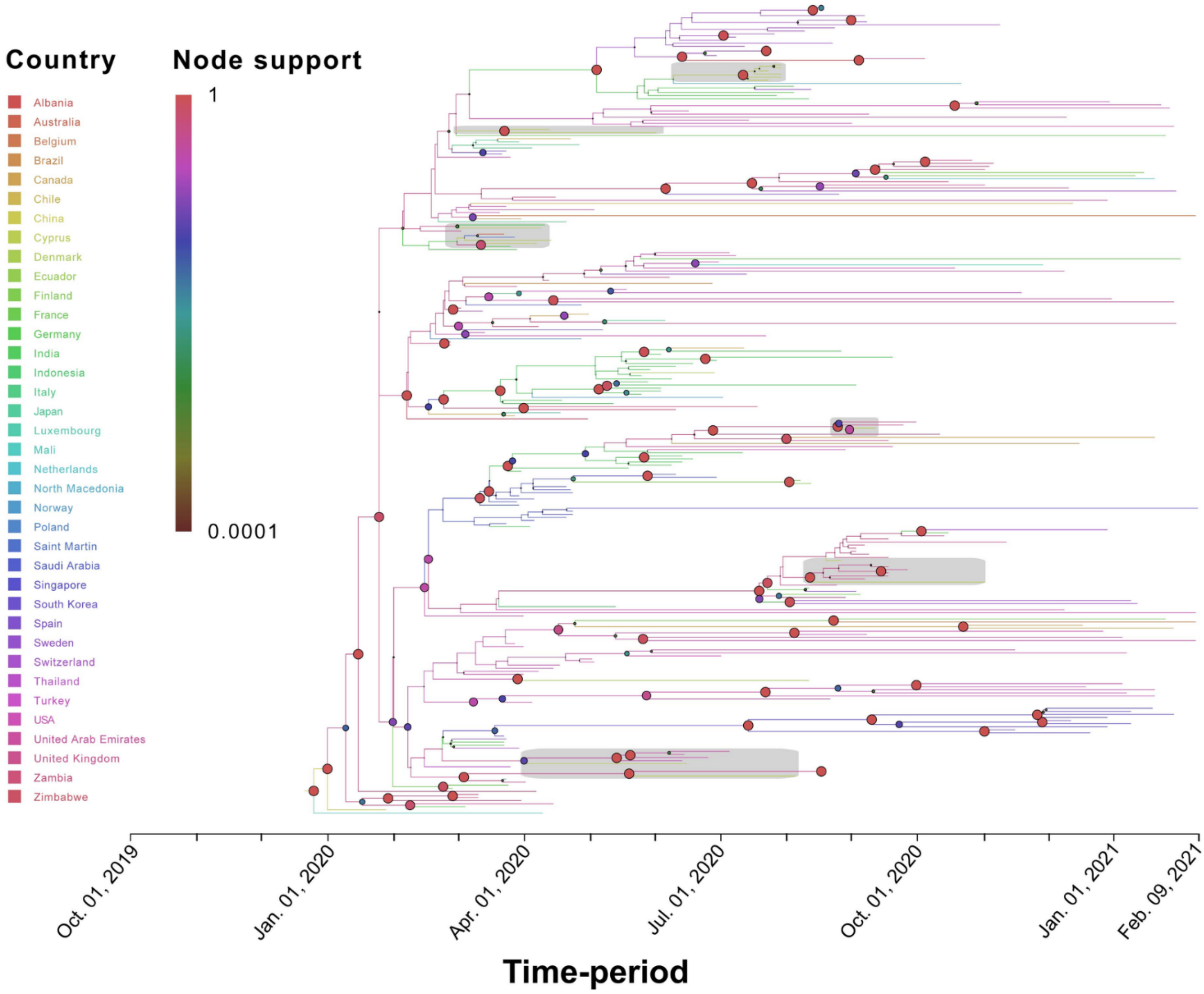


**Figure S3. Time-scaled migration history for the B.basal dataset.** The link between branch colors and the inferred location (Country) is shown in the top left of the figure. The size and color of the circles at the nodes (representing inferred common ancestors) indicate their posterior support (node support); the level of node support (highest = 1 and lowest = 0.0001) corresponds to the size of the circle (higher support = larger circle size). The link between color and posterior node support is shown in the top left of the figure. The gray-shaded boxes indicate the Cypriot samples. The x-axis representing the time-period of the reconstruction was generated by the maximum clade credibility summary tree.

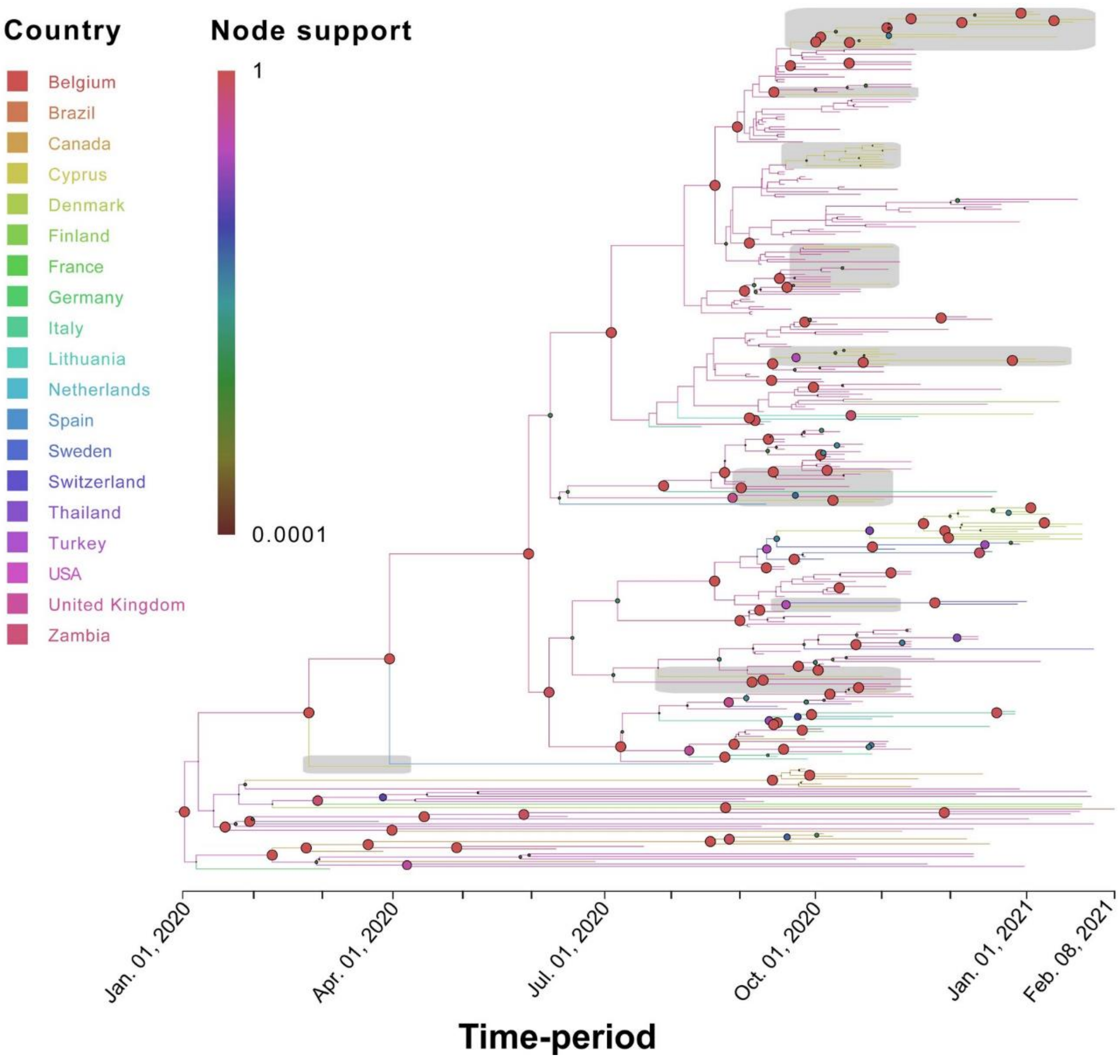


**Figure S4. Time-scaled migration history for the B.1.177 dataset.** The link between branch colors and the inferred location (Country) is shown in the top left of the figure. The size and color of the circles at the nodes (representing inferred common ancestors) indicate their posterior support (node support); the level of node support (highest = 1 and lowest = 0.0001) corresponds to the size of the circle (higher support = larger circle size). The link between color and posterior node support is shown in the top left of the figure. The gray-shaded boxes indicate the Cypriot samples. The x-axis representing the time-period of the reconstruction was generated by the maximum clade credibility summary tree.

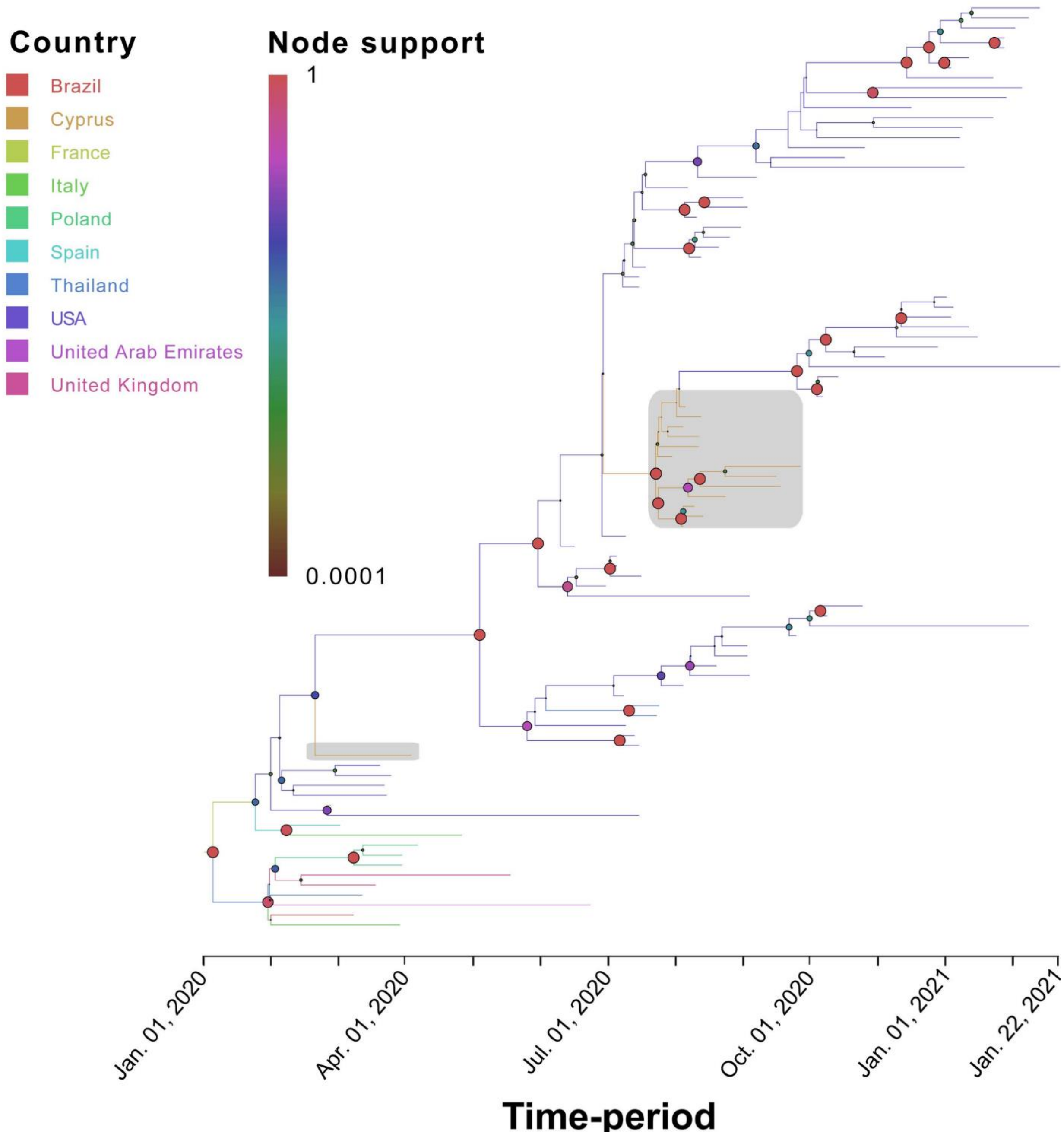


**Figure S5.** Time-scaled migration history for the B.1.2 dataset. The link between branch colors and the inferred location (Country) is shown in the top left of the figure. The size and color of the circles at the nodes (representing inferred common ancestors) indicate their posterior support (node support); the level of node support (highest = 1 and lowest = 0.0001) corresponds to the size of the circle (higher support = larger circle size). The link between color and posterior node support is shown in the top left of the figure. The gray-shaded boxes indicate the Cypriot samples. The x-axis representing the time-period of the reconstruction was generated by the maximum clade credibility summary tree.

**Table S4.** The estimated number of migration events towards and from Cyprus for each dataset. Lower and upper refer to the bounds of the 95% HPD interval.

| | **From[1]** | | |
|---|---|---|---|
| **Countries/Subregions[2]** | **Average[3]** | **Lower[4]** | **Upper[5]** |
| United Kingdom | 129.8 | 47 | 194 |
| Southern Europe | 6.5 | 0 | 13 |
| Germany | 4 | 0 | 13 |
| Eastern Europe | 2.4 | 0 | 8 |
| USA | 2 | 1 | 3 |
| Italy | 1.4 | 0 | 5 |
| South Eastern Asia | 1.4 | 0 | 5 |
| Slovenia | 1 | 0 | 2 |
| | **To[6]** | | |
| United Kingdom | 69.7 | 15 | 113 |
| USA | 1.7 | 1 | 5 |
| Chile | 1.1 | 0 | 2 |
| Finland | 1 | 1 | 1 |
| Jordan | 0.9 | 0 | 2 |
| Czech Republic | 0.8 | 0 | 3 |
| Switzerland | 0.7 | 0 | 2 |
| Pakistan | 0.5 | 0 | 1 |
| Brazil | 0.3 | 0 | 1 |
| Denmark | 0.3 | 0 | 2 |

[1] "From" indicates the migration events of a country/subregion from which migration events were initiated from.

[2] Countries/subregions are as denoted by United Nations geographical subregion.

[3,4,5] Represent average Markov jumps based on the lower and upper bounds of the of the 95% HPD interval migration events towards and from Cyprus.

[6] "To" indicates the migration events from a country/subregion from which migration events were directed to.

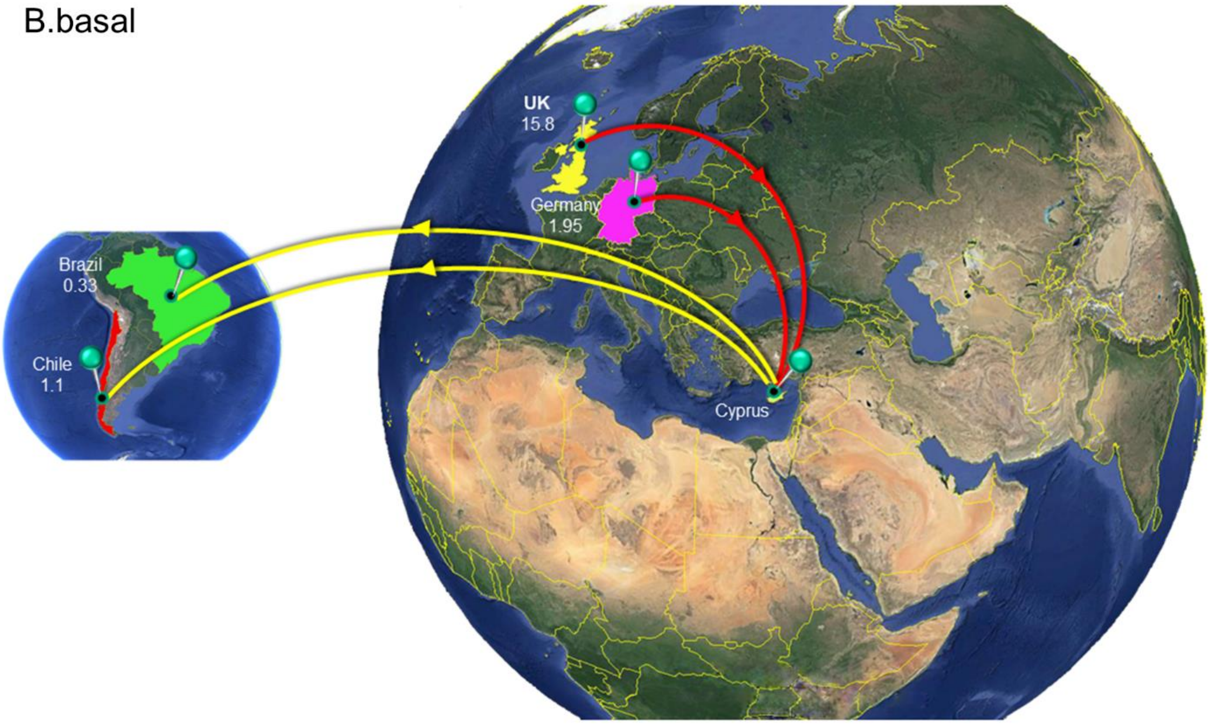


**Figure S6.** Map of SARS-CoV-2 B.basal clade group transmission between Cyprus and other countries. The geographic origins of the SARS-CoV-2 B.basal clade imported into Cyprus based on the statistical phylogeographic analysis are shown as red lines, and exports from Cyprus to other countries are shown as yellow lines. Countries acting as “sources” or “sinks” for SARS-CoV-2 B.basal transmission are highlighted and labeled, and the average number of migration events is indicated. Map images courtesy of Google Earth Pro 7.3.2.5776 (December 14, 2015). Center: Global view centered on Europe. 36°16′38.78″N 36°07′29.71″E, Eye alt 7949.12 km. US Dept. of State Geographer, DATA SIO, NOAA, U.S. Navy, NGA, GEBCO. Image Landsat/Copernicus. 2018 © Google. Left: Brazil and Chile. 26°53’48.58” S 60°23’04.07” W, Eye alt 10919.25 km. Image Landsat/Copernicus, US Dept. of State Geographer, Data SIO, NOAA, U.S. Navy, NGA, GEBCO, © 2021 Google https://www.google.com/earth/versions/#earth-pro [April 10, 2019 and March 23, 2021].

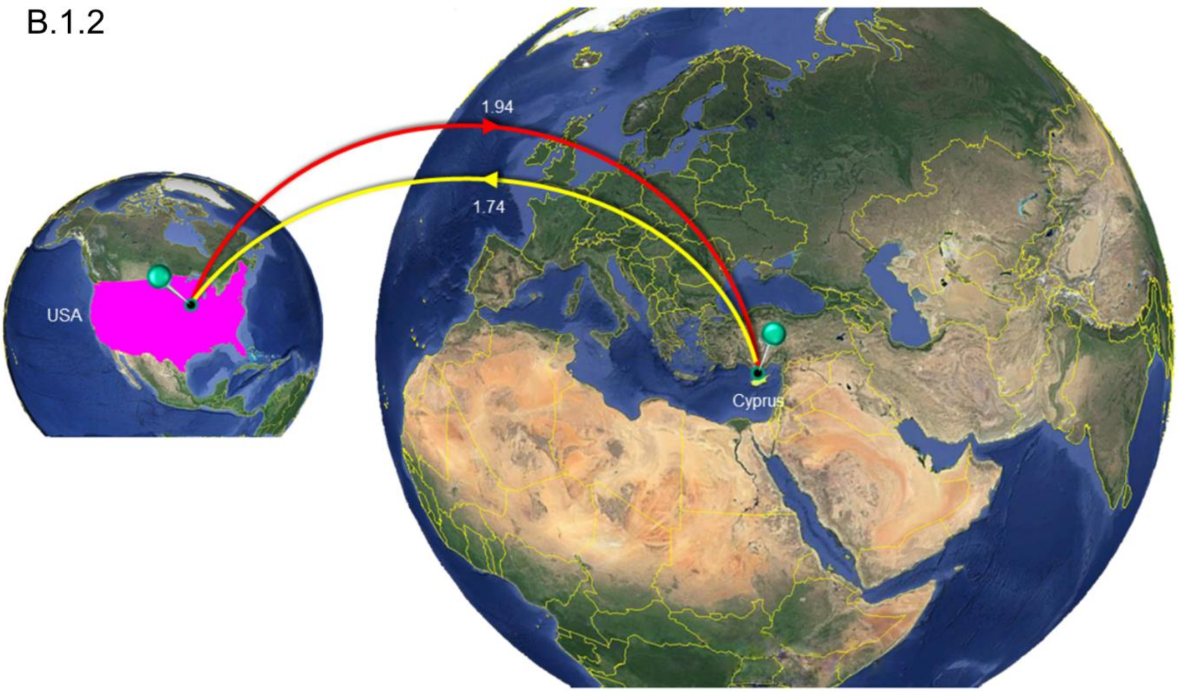


**Figure S7.** Map of SARS-CoV-2 B.1.2 clade group transmission between Cyprus and other countries shows imports from and exports to only the United States. The geographic origins of SARS-CoV-2 B.basal imported into Cyprus based on the statistical phylogeographic analysis are shown as red lines, and export from Cyprus to the United States is shown as a yellow line. The two countries that act as "sources" and "sinks" for SARS-CoV-2 B.1.2 transmission are highlighted and labeled, and the average number of migration events is indicated. Map images courtesy of Google Earth Pro 7.3.2.5776 (December 14, 2015). Center: Global view centered on Europe. 36°16′38.78″N 36°07′29.71″E, Eye alt 7949.12 km. US Dept. of State Geographer, DATA SIO, NOAA, U.S. Navy, NGA, GEBCO. Image Landsat/Copernicus. 2018 © Google. Left: United States of America. 39°14′14.35″N 97°53′40.02″W, Eye alt 7891.99 km. DATA SIO, NOAA, U.S. Navy, NGA, GEBCO. 2019 © GeoBasis-DE/BKG. https://www.google.com/earth/versions/#earth-pro [April 10, 2019 and March 23, 2021].

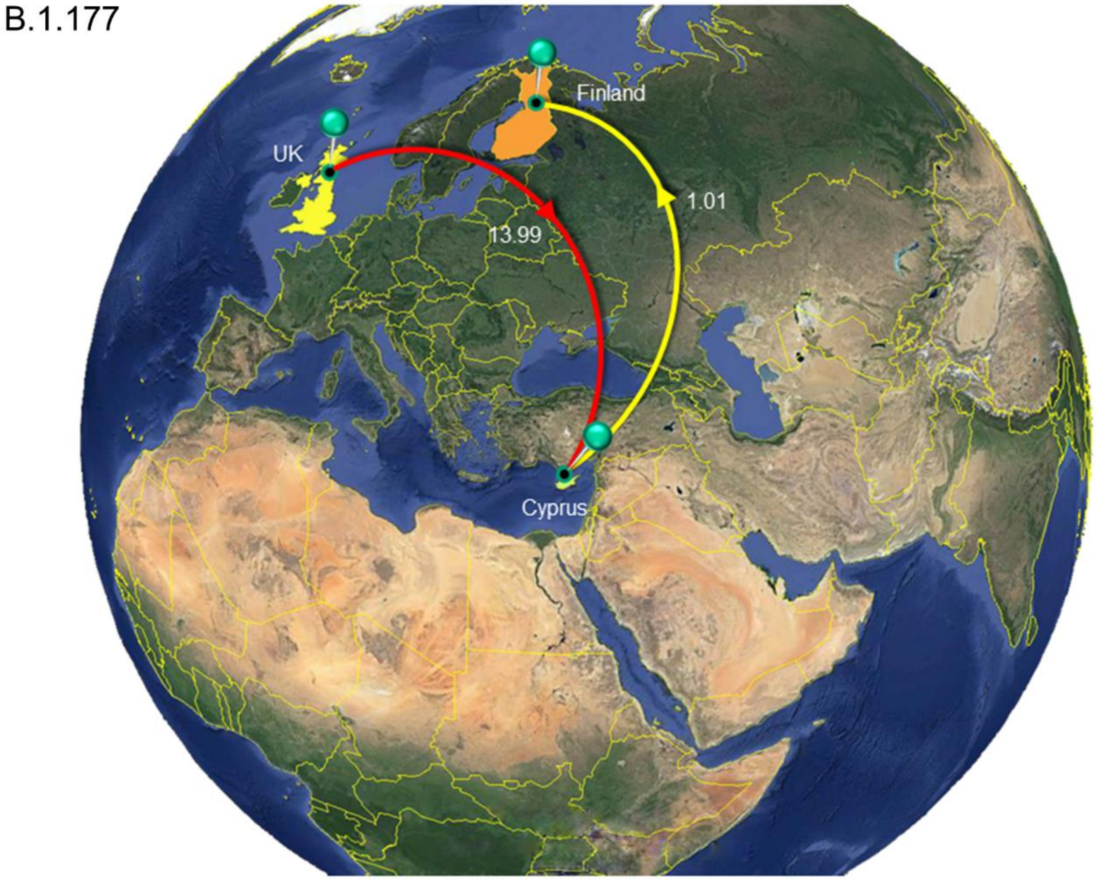


**Figure S8.** Map of SARS-CoV-2 B.1.177 clade group transmission between Cyprus and other countries. The geographic origins of SARS-CoV-2 B.1.177 imported into Cyprus based on the statistical phylogeographic analysis are shown as red lines, and exports from Cyprus to other countries are shown as yellow lines. Countries acting as “sources” or “sinks” for SARS-CoV-2 B.basal transmission are highlighted and labeled, and the average number of migration events is indicated. Map images courtesy of Google Earth Pro 7.3.2.5776 (December 14, 2015). Global view centered on Europe. 36°16′38.78″N 36°07′29.71″E, Eye alt 7949.12 km. US Dept. of State Geographer, DATA SIO, NOAA, U.S. Navy, NGA, GEBCO. Image Landsat/Copernicus. 2018 © Google. https://www.google.com/earth/versions/#earth-pro [April 10, 2019].

## References

1. Rambaut, A.; Lam, T.T.; Carvalho, L.M.; Pybus, O.G. Exploring the temporal structure of heterochronous sequences using TempEst (formerly Path-O-Gen). *Virus Evol.* **2016**, *2*, doi:10.1093/ve/vew007.
